# Towards ferroelectricity-inducing chains of halogenoantimonates(iii) and halogenobismuthates(iii)†

**DOI:** 10.1039/d0ra10151f

**Published:** 2021-05-13

**Authors:** Magdalena Owczarek, Przemysław Szklarz, Ryszard Jakubas

**Affiliations:** Faculty of Chemistry, University of Wroclaw F. Joliot Curie 14 50-383 Wroclaw Poland magdalena@lanl.gov

## Abstract

In halogenoantimonate(iii) and halogenobismuthate(iii) organic–inorganic hybrids, chains of *trans*-connected octahedra, *trans*-[MX_5_]_∞_, are considered attractive anionic structures for inducing ferroelectricity. The latter is realized by displacing the bridging halogen atoms along the chain direction – the process that changes the polarity of the whole unit. Advances in the identification of such materials have been hindered, however, by substantial difficulty in obtaining such structures. Here we investigate structural and dielectric properties of three families of compounds based on 2-mercaptopyrimidinium, 2-aminopyrimidinium, and 2-amino-4-methylpyrimidinium cations in which 8 out of 12 compounds show *trans*-[MX_5_]_∞_ chains in their crystal structures. Two of the compounds adopt a polar *P*2_1_ space group and are potentially ferroelectric. We perform a detailed structural analysis of all compounds with *trans*-[MX_5_]_∞_ chains discovered by far to understand the factors that lead to the chains' formation. We reveal that the size of a cation predominantly defines the accessibility of structures with this anionic form and we provide rules for designing hybrids with *trans*-[MX_5_]_∞_ chains to help guide future efforts to engineer materials with interesting non-linear electrical properties.

## Abstract 2

A discovered abundance of structures with rare and highly-desired anionic chains is examined to identify structural factors leading to the chains' formation.

## Graphical abstract

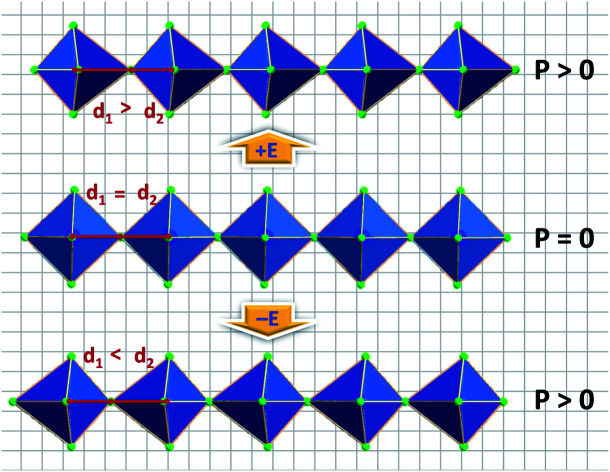

## Introduction

Organic–inorganic hybrids composed of metal halide frameworks and organic cations have been extensively studied for the last few decades.^1–5^ This tremendous interest in exploring these systems is driven by the identification of an impressive number of materials with excellent luminescent,^6,7^ thermochromic,^8,9^ or optoelectronic^10–12^ properties. Coupled with their structural tunability, low production costs, and room-temperature processability, the organic–inorganic hybrids have been recognized as promising active materials for technological applications such as solar cells,^13,14^ light-emitting diodes,^15^ and photodetectors.^16,17^ While there is an observable expansion of the hybrids field in regards to the choice of a metal center,^18,19^ the metal ions of groups 14 (Pb^2+^, Sn^2+^) and 15 (Sb^3+^, Bi^3+^) remain in the focus of material chemists and crystal engineers. Among these two groups of ions, halogenoantimonates(iii) and halogenobismuthates(iii) of the general formula of R_*a*_M_*b*_X_3*b*+*a*_ (R – an organic monocation, M = Sb^3+^ or Bi^3+^ and X = Cl^−^, Br^−^, I^−^) represent an extremely active area of research due to the identification of an impressively high number of compounds exhibiting ferroelectric properties.^20,21^ The tendency of the primary building block—MX_6_^3−^ octahedron—to share up to four ligands with other octahedra leads to the observation of an astonishing variety of anionic units; by now, more than 40 types have been recognized. Among them, the ones that have been found in the ferroelectric systems are: (i) zero-dimensional (0D) corner-sharing M_2_X_11_^5−^ bioctahedron,^22–26^ (ii) 0D face-sharing M_2_X_9_^3−^ bioctahedron,^27–30^ (iii) 2D ‘honeycomb’ M_2_X_9_^3−^ structure,^31–34^ (iv) 1D [MX_4_]_∞_ chain,^35^ and (v) 1D [MX_5_]_∞_ chain.^36–45^ While (i)–(iv) ferroelectric cases are well known by now, particularly interesting is the recent significant boost in the number of reported ferroelectric materials with [MX_5_]_∞_ chains (v).

In general, within halogenoantimonates(iii) and halogenobismuthates(iii) with amine : metal ratio 2 : 1 (R_2_MX_5_ stoichiometry), only 0D or 1D anionic units have been identified. The 0D group comprises of isolated square pyramidal MX_5_^2−^ units, M_2_X_10_^4−^ bioctahedral units, and M_4_X_20_^8−^ four-octahedral units. Among the 1D units, chains of *trans*-connected MX_6_^3−^ octahedra (∼180° X_bridging_–M–X_bridging_ angle) and *cis*-connected MX_6_^3−^ octahedra (∼90° X_bridging_–M–X_bridging_ angle) can be distinguished. In 2012,^46^ we performed a structural survey, recently updated by Ksiądzyna *et al.*,^44^ of R_2_MX_5_ compounds deposited in the Cambridge Structural Database to find a relationship between the acentric symmetry of the compounds and the type of anionic unit present in a crystal. The results of the survey indicated that infinite 1D chains lead more frequently to the acentric arrangement (40% of cases) than 0D units (up to 10% of cases). Responsible for this statistic seems to be much greater ease of deformation of the 1D chains in comparison to the highly-symmetric 0D units that locate themselves around symmetry centers in a crystal.^46^ Therefore, it was anticipated to see much more ferroelectric R_2_MX_5_ systems in the future than the three ferroelectrics known at that time: (MV)BiI_3_Cl_2_ and (MV)BiBr_5_ where MV is methylviologen dication,^36,37^ and (C_3_N_2_H_5_)_2_SbCl_5_ with imidazolium cation.^38^ Indeed, in the last several years, seven new ferroelectric compounds^39–45^ with 1D [MX_5_]_∞_ chains were discovered. In all of them, the deformation of the chains gives a significant contribution to the mechanism of paraelectric-to-ferroelectric transitions. Noteworthy is the fact that these new ferroelectrics are characterized by the presence of *cis*-[MX_5_]_∞_ chains, leaving (MV)BiI_3_Cl_2_ and (MV)BiBr_5_ as the only ferroelectrics with chains of *trans*-connected octahedra.^36,37^ Among the latter compounds, special attention should be paid in particular to (MV)BiI_3_Cl_2_ as its spontaneous polarization value (80 μC cm^−2^ in the first field sweep, ∼15 μC cm^−2^ subsequent sweeps) is one of the highest among ferroelectric organic–inorganic hybrids.^37^ Since polarization switching in this material is achieved along the *trans*-[MX_5_]_∞_ chains, a displacive mechanism related to a movement of bridging halogen atoms is postulated. A similar mechanism is known to be responsible for the polarization switching in pure inorganic materials from the perovskite family, *e.g.*, PZT and PLZT,^47^ which are most studied because of their robustness and practical applications. Easily deformable *trans*-[MX_5_]_∞_ chains of halogenoantimonates(iii) and halogenobismuthates(iii) might, therefore, open an avenue for the development of organic–inorganic materials with the values of polarization similar to the ones obtained by inorganic ferroelectrics. The obstacle here is the fact that *trans*-[MX_5_]_∞_ chains have been rarely observed and, by far, only a few organic cations have been found to be engaged in such systems: methylviologen dication,^36,37,48^ 2-chloropyridinium cation,^49^ ethyldimethylammonium cation,^50^ and 2-mercaptopyrimidinium cation^51^ (bromoantimonate(iii) only). To enlarge this database of compounds and to shed more light on the factors that might lead to the formation of this particular chain configuration, we explored further the incorporation of pyrimidinium derivatives into halogenoantimonates(iii) and halogenobismuthates(iii) networks. We started by completing the 2-mercaptopyrimidine (2Sprm) family by obtaining the missing chloride and bromide analogs and subsequently moved to 2-aminopyrimidine (2Aprm) and 2-amino-4-methylpyrimidine (2A4Mprm). In 8 out of 12 compounds, the amines produced structures with the desired *trans*-[MX_5_]_∞_ chains. Different sizes of the cations and a variety of functional groups influencing intermolecular interactions make these results of particular value in terms of the identification of structural parameters, analyzed in the Discussion section, that should be taken under consideration when designing new systems.

## Experimental

All starting materials were used as received: 2-mercaptopyrimidine (98%, Sigma Aldrich), 2-aminopyrimidine (97%, Sigma Aldrich), 2-amino-4-methylpyrimidine (97%, Sigma Aldrich), Sb_2_O_3_ or Bi_2_O_3_ (≥99%, Sigma Aldrich), hydrochloric acid (37%, Sigma Aldrich), hydrobromic acid (48%, Sigma Aldrich). The complexes were prepared by dissolving a stoichiometric amounts of an amine with antimony(iii) or bismuth(iii) oxide in hot HCl or HBr. After cooling the mixtures to ambient temperature, they were placed in a desiccator with KOH pellets used as a desiccant. Within two weeks, crystals of target materials appeared. Elemental analyses: (2Sprm)_2_SbCl_5_: 18.31% C, 10.50% N, 1.84% H (calc. 18.29% C, 10.66% N, 1.92% H); (2Sprm)_2_SbBr_5_: 12.93% C, 7.30% N, 1.28% H (calc. 12.85% C, 7.49% N, 1.35% H); (2Sprm)_2_BiCl_5_: 15.69% C, 9.03% N, 1.57% H (calc. 15.69% C, 9.15% N, 1.65% H); (2Sprm)_2_BiBr_5_: 11.46% C, 6.53% N, 1.18% H (calc. 11.51% C, 6.71% N, 1.21% H); (2Aprm)_2_SbCl_5_: 19.59% C, 17.02% N, 2.40% H (calc. 19.56% C, 17.11% N, 2.46% H); (2Aprm)_2_SbBr_5_: 13.46% C, 11.78% N, 1.58% H (calc. 13.47% C, 11.78% N, 1.70% H); (2Aprm)_4_Bi_2_Cl_10_:16.05% C, 14.13% N, 2.31% H (calc. 16.11% C, 14.09% N, 2.37% H); (2Aprm)_4_Bi_2_Br_10_: 12.07% C, 10.80% N, 1.36% H (calc. 12.00% C, 10.50% N, 1.51% H); (2A4Mprm)_2_SbCl_5_·H_2_O: 22.30% C, 15.52% N, 3.42% H (calc. 22.35% C, 15.64% N, 3.38% H); (2A4Mprm)_2_BiCl_5_·H_2_O: 19.26% C, 13.41% N, 2.81% H (calc. 19.23% C, 13.46% N, 2.90% H); (2A4Mprm)SbBr_5_·H_2_O: 9.17% C, 6.49% N, 1.74% H (calc. 9.23% C, 6.46% N, 1.70% H); (2A4Mprm)_4_Bi_2_Br_10_: 14.40% C, 10.00% N, 1.87% H (calc. 14.49% C, 10.14% N, 1.95% H).

The X-ray diffraction data were collected at 100 K, or 120 K for (2Aprm)_4_Bi_2_Cl_10_, using Oxford Diffraction Xcalibur diffractometer with Onyx or Sapphire2 CCD detectors and Mo Kα radiation. Data collection and reduction were carried out with CrysAlis CCD and CrysAlis PRO. Refinement details for each family of compounds, selected bonds and angles values, and hydrogen bond parameters can be found in ESI (Tables S1–S9†).

Simultaneous thermogravimetric analysis (TGA) and differential thermal analysis (DTA) were performed using a Setaram SETSYS 16/18 instrument between 300 and up to 875 K (Fig. S1–S3†) with a ramp rate of 2 K min^−1^. The scans were performed under flowing nitrogen (flow rate: 1 dm^3^ h^−1^). PerkinElmer 8500 Differential Scanning Calorimeter (DSC), calibrated using *n*-heptane and indium, was used to analyze the thermal stability of the compounds between 100 K and up to 20 K below the materials' melting point. Hermetically sealed Al pans with the polycrystalline material were prepared in a controlled-atmosphere N_2_ glovebox.

The frequency dependence of complex electric permittivity ε* = *ε*′ − i*ε*′ was measured with an Agilent 4980A Precision LCR Meter in the frequency range 100 Hz to 2 MHz. The dimensions of the samples used in these measurements were approximately 4 × 4 × 1 mm^3^. In all cases, silver electrodes were applied on crystal's widest faces. The overall error in electric permittivity measurements was less than 5%.

## Results

### 2-Mercaptopyrimidinium family

**Fig. 1:**
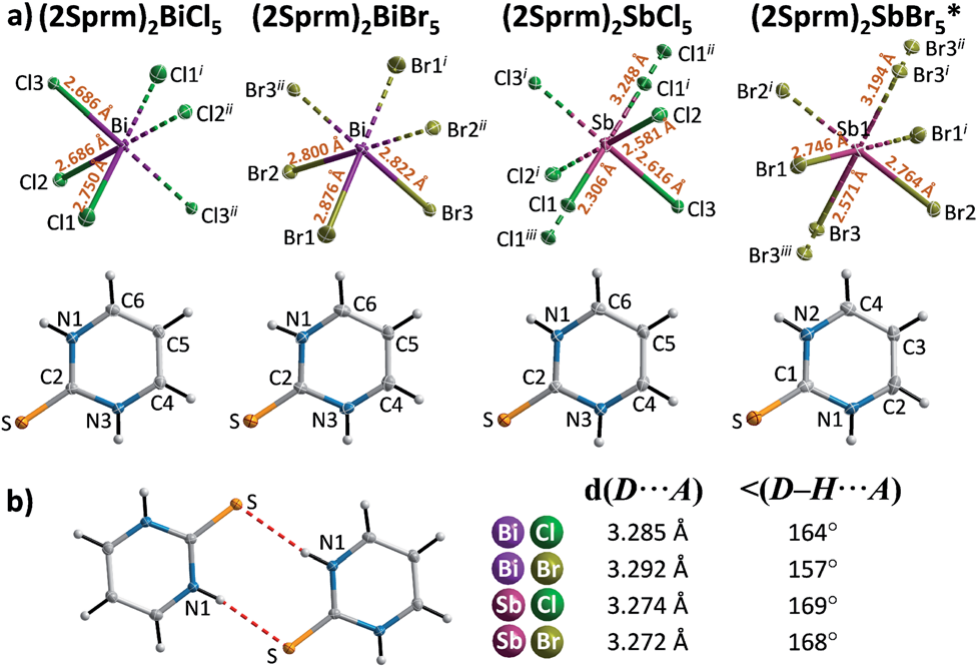
(a) The asymmetric part of the unit cell and the atom numbering scheme of 2-mercaptopyrimidinium crystals. Thermal ellipsoids are shown at the 50% probability level. Dashed two-colored lines correspond to symmetry related atoms. * Crystal structure reported by Ozturk *et al.*^51^ (b) A cationic dimer with geometrical parameters of its N–H⋯S hydrogen bonds.

**Fig. 2:**
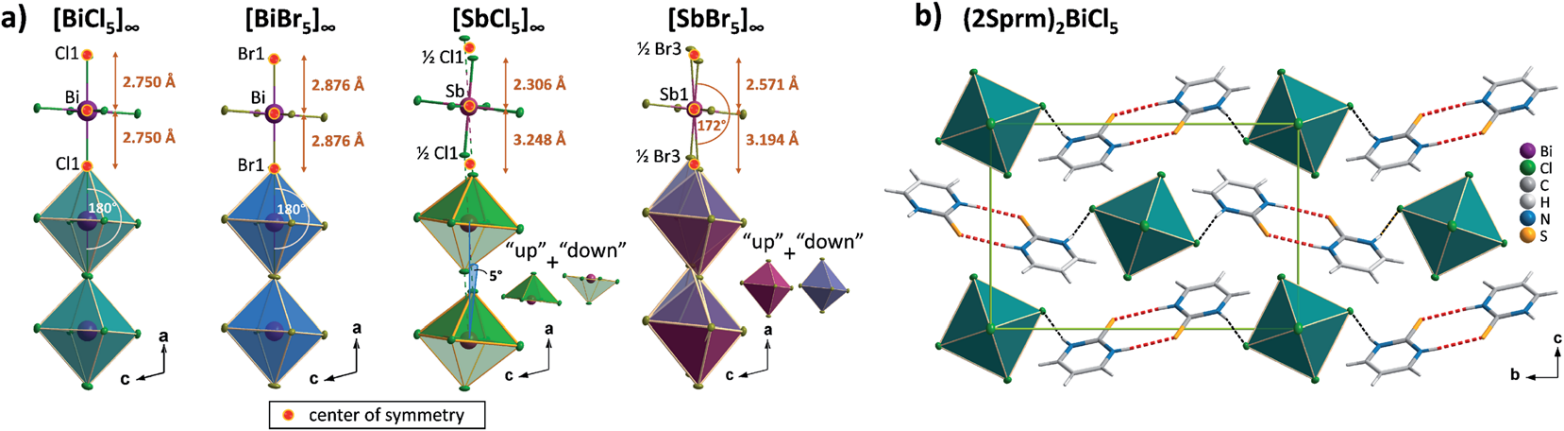
(a) Anionic units observed in the 2-marcaptopyrimidinium crystals with selected geometrical parameters shown. (b) Unit cell packing of (2Sprm)_2_BiCl_5_ along the *a* axis.

Four members of (2Sprm)_2_MX_5_ family (M = Sb^3+^, Bi^3+^; X = Cl^−^, Br^−^) were easily obtained *via* a standard procedure (see Experimental section). No solid-state phase transitions were detected by Differential Scanning Calorimetry (DSC). Structural characterization revealed that the three new analogs with 2-mercaptopyrimidinium cation, (2Sprm)_2_BiCl_5_, (2Sprm)_2_BiBr_5_, and (2Sprm)_2_SbCl_5_, are isomorphous with the already reported (2Sprm)_2_SbBr_5_ (Fig. 1a) and crystallize in centrosymmetric *P*2_1_/*n* space group (Table 1). Each of the crystal structures composes of 2-mercaptopyrimidinium cations in their thione tautomeric – forming dimeric units *via* N–H⋯S hydrogen bonds of approx. 3.28 Å in distance and 157°–169° angle (Fig. 1b) – and more or less distorted octahedral MX_6_^3−^ units. In (2Sprm)_2_BiCl_5_ and (2Sprm)_2_BiBr_5_, the MX_6_^3−^ units are joined *via* X1 halogen atoms and form one-dimensional chains of *trans*-connected octahedra (180° X1–M–X1 angle; Fig. 2a). The chains are considered apolar due to the positioning of metal centers and bridging ligands in special positions (the centers of symmetry) which determines equal M–X1 and M–X1^*i*^ distances. In the case of antimonate(iii) analogs, the bridging halogen atoms are disordered between two positions with 0.5 occupancy each, which differentiates the M–X_bridging_ distances: 2.306 and 3.248 Å in (2Sprm)_2_SbCl_5_, and 2.571 and 3.194 Å in (2Sprm)_2_SbBr_5_. Such formed 1D anionic structure can be considered a superimposition of two polar, oppositely-oriented *trans*-[SbBr_5_]_∞_ chains—or *pseudo-trans*-[SbCl_5_]_∞_ chains with 3.248 Å distance not being considered as a chemical bond—giving as a result apolar 1D chains. The crystal packing (Fig. 2b) resembles a chessboard arrangement when viewed along the *a*-axis direction. The organic 2Sprm^+^ dimers are located between the inorganic units and are stabilized by N–H⋯X hydrogen bonds of moderate strength (3.2–3.4 Å donor–acceptor distance and approx. 170° angle) connecting them to the inorganic network.

**Table 1:** Selected crystal data and structure refinement parameters of 2Sprm and 2Aprm crystals. * Crystal structure reported by Ozturk *et al.*^51^

Crystal	(2Sprm)_2_BiCl_5_	(2Sprm)_2_BiBr_5_	(2Sprm)_2_SbCl_5_	(2Sprm)_2_SbBr_5_*	(2Aprm)_2_SbCl_5_	(2Aprm)_2_SbBr_5_	(2Aprm)_4_Bi_2_Cl_10_·2H_2_O	(2Aprm)_4_Bi_2_Br_10_
Empirical formula	C_8_H_10_N_4_SBiCl_5_	C_8_H_10_N_4_SBiBr_5_	C_8_H_12_N_6_SbCl_5_	C_8_H_12_N_6_SbBr_5_	C_8_H_12_N_6_SbCl_5_	C_8_H_12_N_6_SbBr_5_	C_16_H_28_N_12_O_2_Bi_2_Cl_10_	C_8_H_12_N_6_BiBr_5_
Formula weight/g mol^−1^	612.55	834.85	525.32	747.60	491.24	713.54	1192.96	800.77
Temperature/K	100(2)	100(2)	100(2)	100(2)	100(2)	100(2)	120(2)	100(2)
Crystal system	Monoclinic	Monoclinic	Monoclinic	Monoclinic	Monoclinic	Monoclinic	Monoclinic	Triclinic
Space group	*P*2_1_/*n*	*P*2_1_/*n*	*P*2_1_/*n*	*P*2_1_/*n*	*P*2_1_	*P*2_1_	*C*2/*c*	*P*1̄
*a*/Å	5.500(4)	5.752(4)	5.540(4)	5.7512(1)	5.713(3)	5.946(3)	19.226(4)	7.873(3)
*b*/Å	15.082(5)	15.263(5)	14.910(5)	15.1339(4)	14.125(5)	14.401(4)	13.018(4)	11.404(4)
*c*/Å	10.360(4)	10.633(4)	10.210(5)	10.5575(3)	10.254(4)	10.621(4)	14.193(4)	11.618(4)
*α*	90.00	90.00	90.00	90.00	90.00	90.00	90.00	115.80(5)
*β*	104.39(2)	104.77(2)	104.08(2)	104.248(3)	105.65(2)	105.31(2)	100.74(2)	96.30(4)
*γ*	90.00	90.00	90.00	90.00	90.00	90.00	90.00	98.86(4)
*V*/Å^3^	832.4(7)	902.7(8)	818.0(8)	890.64(4)	796.8(6)	977.2(6)	3490.1(16)	909.4(7)
*Z*	2	2	2	2	2	2	4	2
Goodness-of-fit on *F*^2^	1.02	1.03	1.05	0.97	1.08	1.10	0.97	1.04
Flack parameter	—	—	—	—	0.397(14)^a^	0.446(13)^a^	—	—
CCDC number	2023490	2023491	2023489	702618	2025386	2025387	2025388	2025389

a Racemic twin.

**Fig. 3:**
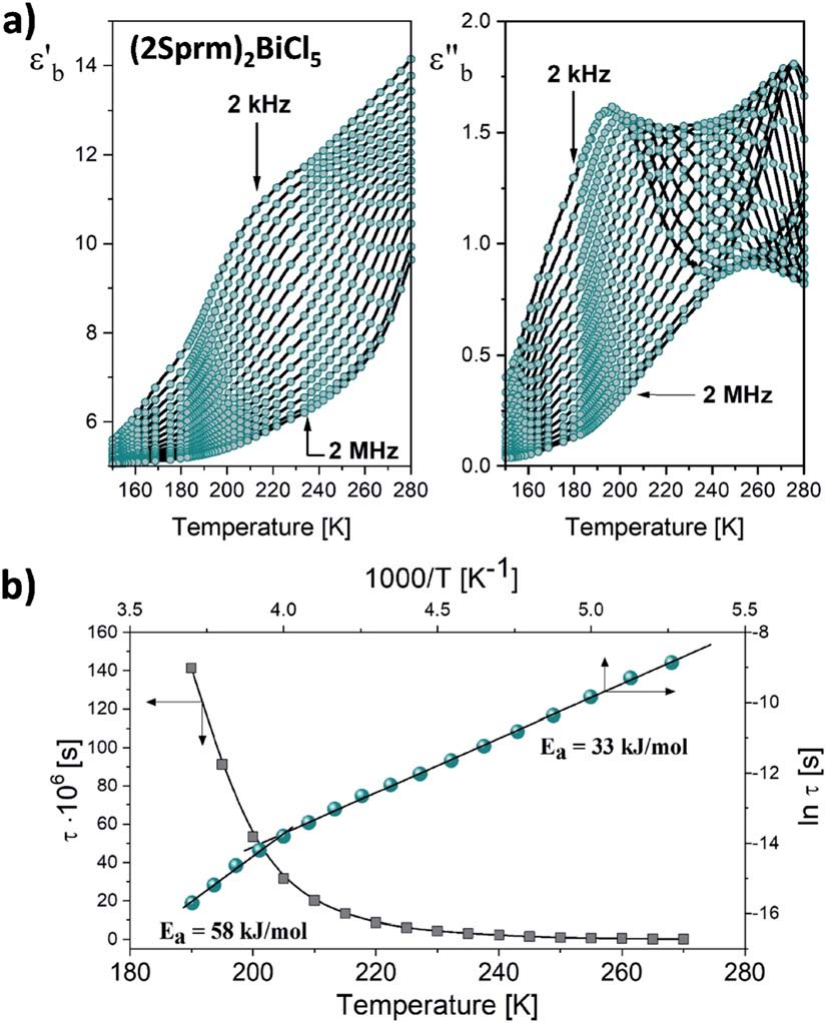
(a) Temperature-dependent real and imaginary parts of the complex electric permittivity obtained on cooling (2Sprm)_2_BiCl_5_ crystal. (b) *τ vs. T* and ln *τ vs.* 1000/*T* plots along with the estimated values of the activation energy.

Dynamic relaxation processes detected below room temperature in the dielectric response of the crystalline samples suggest substantial freedom of movement of the polar organic moieties, in particular in bismuth complexes with larger voids and weaker intermolecular interactions. Fig. 3a presents the temperature dependence of the real and imaginary parts of the complex electric permittivity obtained along the crystallographic *b* axis of (2Sprm)_2_BiCl_5_ crystal. The response, measured for 2 kHz–2 MHz frequency range, can be well described by the Cole–Cole relation:1
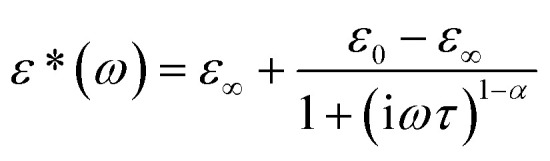
where *ε*_0_ and *ε*_∞_ are low and high frequency limits of the electric permittivity, respectively, *ω* is an angular frequency, *τ* is macroscopic relaxation time. The determined parameters of eqn (1) and Cole–Cole diagrams for selected temperatures are presented in Fig. S4 and Table S10.† The *α* values ranging between 0.4 and 0.5 suggest the polydispersive nature of the observed relaxation process arising from the presence of at least two relaxators with similar dynamics. Indeed, two values of the activation energy (33 kJ mol^−1^ for the low-temperature region and 58 kJ mol^−1^ for the high-temperature region; Fig. 3b), estimated using the Arrhenius relation for the macroscopic relaxation time, 
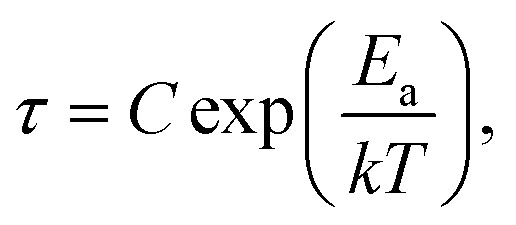
 confirm the contribution of two relaxators to the relaxation process and their high values suggest the movement of large polar organic units.

### 2-Aminopyrimidinium family

**Fig. 4:**
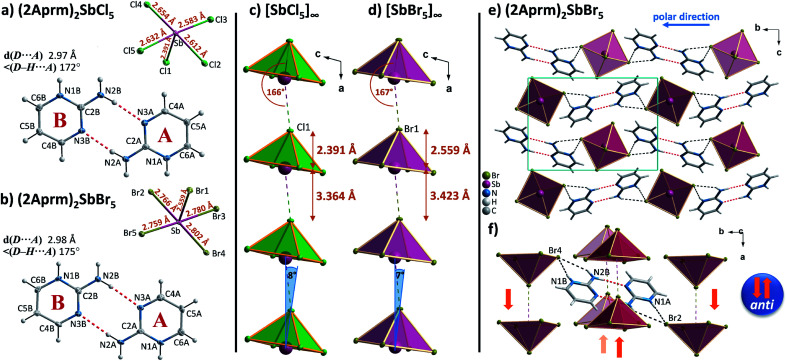
The asymmetric part of the unit cell and the atom numbering scheme of (2Aprm)_2_SbCl_5_ (a) and (2Aprm)_2_SbBr_5_ (b) at 100 K. Thermal ellipsoids are shown at the 50% probability level. Anionic *pseudo-trans* chains in (2Aprm)_2_SbCl_5_ (c) and (2Aprm)_2_SbBr_5_ (d). (e) Unit cell packing of (2Aprm)_2_SbBr_5_ along the *a*-axis direction with intermolecular interactions shown as red and black dotted lines. (f) The closest environment of the cationic dimer with the antiparallel orientation of chains shown.

The introduction of a proton-donating group at the second position of pyrimidinium cation finds its consequences in the reduced number of compounds in 2-aminopyrimidinium (2Aprm) family with *trans*-[MX_5_]_∞_ chains. It was found that only antimonate(iii) analogs favor the 1D anionic structure. The analysis of bismuth(iii) analogs revealed the presence of 0D Bi_2_X_10_^4−^ bioctahedral units. The two halogenoantimonates(iii) compounds, (2Aprm)_2_SbCl_5_ and (2Aprm)_2_SbBr_5_, are isomorphous and crystallize in a polar *P*2_1_ space group (Table 1); no structural transitions were detected with DSC. The acentric symmetry was indirectly confirmed by the observation of characteristic anomalies in electric permittivity (Fig. S5†) arising from the resonant piezoelectric contribution.^46,52,53^ The asymmetric parts of the unit cells consist of one SbX_5_^2−^ square pyramidal unit and two nonequivalent 2Aprm^+^ cations, marked as A and B in Fig. 4a and b, forming dimers *via* N–H⋯N hydrogen bonds of ∼3 Å in distance and angle 172°–175°. The SbX_5_^2−^ pyramids are arranged one above the other (Fig. 4c and d) along the *a*-axis direction in a *pseudo-trans* chain configuration with the sixth Sb–X distance—3.364 Å for Sb⋯Cl1 and 3.423 Å for Sb⋯Br1—being too large to be treated as a chemical bond. Such formed polar 1D anionic structures are additionally distorted by the tilt (7°–8°) in the position of the SbX_5_^2−^ units. Similar to 2-mercaptopyrimidinium family, the crystal packing resembles a chessboard arrangement (Fig. 4e), when viewed along the chains, with cationic dimers stabilized by N–H^+^⋯Br (3.15–3.35 Å; ∼164°) and N–H⋯Br (3.50–3.70 Å; ∼144°) contacts (Table S6†). Related by a 2-fold screw axis, the polar *pseudo-trans*-[MX_5_]_∞_ chains propagate in opposite directions in the crystal lattice (the *anti* arrangement; Fig. 4f). Nevertheless, the 7°–8° angle disposition of the pyramidal units in each chain gives rise to the polar axis perpendicular to the chains' direction (Fig. 4e). No ferroelectric *P*–*E* hysteresis loop, however, was observed for any of the compounds.

**Fig. 5:**
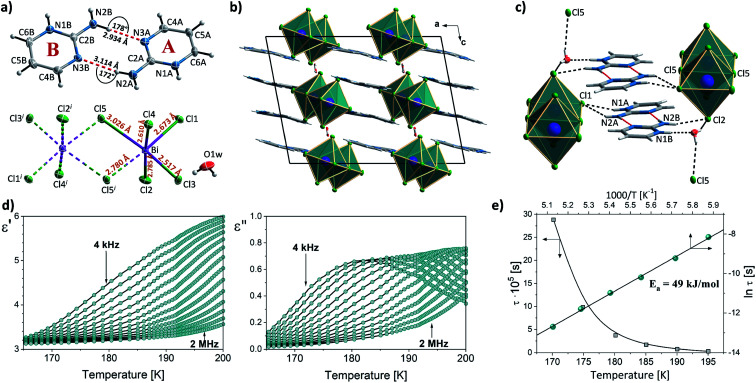
(a) The asymmetric part of the unit cell and the atom numbering scheme of (2Aprm)_4_Bi_2_Cl_10_ at 120 K. Dashed two-colored lines correspond to symmetry-related atoms. Thermal ellipsoids are shown at the 50% probability level. (b) Unit cell packing along the *b*-axis direction. Intermolecular contacts are not shown for clarity. (c) A system of hydrogen bonds linking cationic and anionic substructures. (d) Temperature dependence of the real and imaginary parts of the complex electric permittivity obtained on cooling (2Aprm)_4_Bi_2_Cl_10_ crystal. (e) *τ vs. T* and ln *τ vs.* 1000/*T* plots along with the estimated value of the activation energy.

In combination with chlorobismuthate(iii), 2-aminopyrimidine gives centrosymmetric crystals (*C*2/*c* space group) of (2Aprm)_4_Bi_2_Cl_10_ comprising of two nonequivalent 2Aprm^+^ cations (forming a typical dimer), distorted Bi_2_Cl_10_^4−^ bioctahedral anionic units, and water molecules (Fig. 5a). The dimers are embedded between Bi_2_Cl_10_^4−^ units (Fig. 5b) with their position supported by (i) weak to moderate N–H⋯Cl contacts (A and B cations) of 3.619 and 3.245 Å and 157° and 140° angles, respectively, and (ii) N–H^+^⋯Cl (a cation only) contacts of 3.199 Å and 158°. Water molecules fill the voids next to cations B acting as acceptors of N–H^+^⋯O contacts (2.663 Å, 173°) and donors of two O–H⋯Cl hydrogen bonds with the two closest bioctahedra (Fig. 5c). The uneven strength of intermolecular contacts holding the polar units—2Aprm^+^ and H_2_O molecules—contributes to the appearance of the relaxation process (Fig. 5d) recorded in the dielectric response of (2Aprm)_4_Bi_2_Cl_10_ crystal along the crystal shortest axis. The process is activated around 200 K and can be well described by the Cole–Cole equation (Fig. S6†). Although the obtained α values (0.4–0.45; Table S11†) suggest a strong polydispersive relaxation process, only one value of the energy barrier (49 kJ mol^−1^) has been found indicating very similar dynamics of the relaxators (Fig. 5e). Noteworthy is close to two orders of magnitude increase in the values of the relaxation time, *τ*, within a 25 K temperature range representing substantial slowing down of the motions of the polar units.

**Fig. 6:**
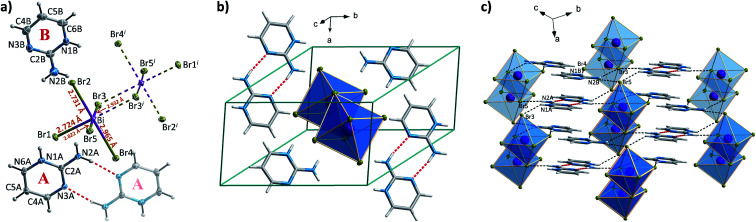
(a) The asymmetric part of the unit cell and the atom numbering scheme of (2Aprm)_4_Bi_2_Br_10_ at 100 K. Dashed two-colored lines correspond to symmetry-related atoms. Thermal ellipsoids are shown at the 50% probability level. (b) Unit cell packing. (c) Intermolecular interactions connecting cationic and anionic substructures.

An unhydrated analog, (2Aprm)_4_Bi_2_Br_10_, crystallizing in the centrosymmetric *P*1̄ space group (Table 1) was obtained with bromobismuthate(iii). Its crystal structure composes of two nonequivalent 2Aprm^+^ cations (A and B in Fig. 6a) and bioctahedral Bi_2_Br_10_^4^. A significant difference in the atomic displacement parameters between the two 2Aprm^+^ cations arises from the cations being engaged in different types of intermolecular contacts. While cation A forms a dimer with another molecule of its type and is stabilized by N–H^+^⋯Br (3.24 Å, 144° angle) and N–H⋯Br (3.65 Å, 141°) hydrogen bonds (Fig. 6b and c), cation B is attached only to the inorganic network by N–H^+^⋯Br (3.3 Å, 154°) and N–H⋯Br (∼3.46 Å, 155°).

### 2-Amino-4-methylpyrimidinium family

**Fig. 7:**
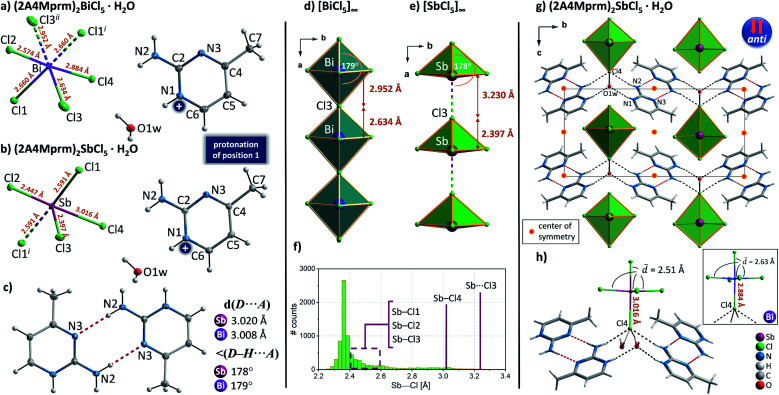
The asymmetric part of the unit cell and the atom numbering scheme of (2A4Mprm)_2_BiCl_5_·H_2_O (a) and (2A4Mprm)_2_SbCl_5_·H_2_O (b) at 100 K. Dashed lines correspond to symmetry-related atoms. Thermal ellipsoids are shown at the 50% probability level. (c) A cationic dimer with geometrical parameters of N–H⋯N hydrogen bonds. Anionic *trans*-mode chains in (2A4Mprm)_2_BiCl_5_·H_2_O (d) and *pseudo-trans* anionic chains in (2A4Mprm)_2_SbCl_5_·H_2_O (e). (f) Distribution of Sb–Cl distances in crystal structures deposited in Cambridge Structural Database with marked positions of Sb–Cl contacts in (2A4Mprm)_2_SbCl_5_·H_2_O. (g) Unit cell packing of (2A4Mprm)2SbCl5·H along the *a*-axis. (h) Main intermolecular contacts in (2A4Mprm)2SbCl5·H2. Inset: A corresponding structural unit in (2A4Mprm)2BiCl5·H.

Complexes with the asymmetrically substituted 2-amino-4-methylpyrimidine (2A4Mprm) showed an unusual ability to accept a proton to more basic nitrogen atom of the ring (position 1), or less basic nitrogen atom (position 3), or both nitrogen atoms (position 1 and 3) – strongly affecting the formation the desired anionic chains. As a result, the presence of *trans*-[MX_5_]_∞_ chains was confirmed in only two cases—(2A4Mprm)_2_BiCl_5_·H_2_O and (2A4Mprm)_2_SbCl_5_·H_2_O. The two compounds are isomorphous and adopt the centrosymmetric *P*2_1_/*m* space group with almost identical unit cell dimensions (Table 2). Their crystal structures consist of 2A4Mprm^+^ cations, water molecules, and either octahedral BiCl_6_^3−^ units or SbCl_5_^2−^ square pyramidal units (Fig. 7a and b). In (2A4Mprm)_2_BiCl_5_·H_2_O, the strongly distorted octahedral units are joined by oppositely-located Cl3 ligands forming 1D *trans*-[BiCl_5_]_∞_ chains that propagate along the *a*-axis direction (Fig. 7d). Substantial deformation of the BiCl_6_^3−^ units is responsible for the polarity of the chains but since the adjacent chains are related to each other *via* centers of symmetry, their direction and, therefore, polarity are opposite (the *anti* arrangement; Fig. 7f). In (2A4Mprm)_2_SbCl_5_·H_2_O, Sb–Cl3 distance (the height of the pyramid) in SbCl_5_^2−^ units is much shorter (2.397 Å) compared to the bismuth compound (2.634 Å). As a result, the distance to the sixth Cl ligand that would complete the octahedral geometry is too large (3.230 Å) to be treated as a chemical bond (Fig. 7f) and a *pseudo-trans* chain configuration is formed. The lack of the sixth ligand allows even stronger deformation of the SbCl_5_^2−^ unit at its base: while three of the Sb–Cl bonds are between 2.45 and 2.60 Å long, the fourth one is elongated up to 3.016 Å – the end value of the acceptable Sb–Cl distances at a spectrum extracted from Cambridge Structural Database (CSD; Fig. 7f). Although Aloui *et al.*^54^ reported the structural data of this compound, collected at room temperature, without accounting the Cl4 atom to the coordination sphere of Sb^3+^ and with a compound's general formula of [C_5_H_8_N_3_]_2_ClSbCl_4_·H_2_O, based on the results of the presented CSD survey we decided to treat Sb–Cl4 distance as a legitimate chemical bond.

**Table 2:** Selected crystal data and structure refinement parameters of 2A4Mprm crystals.

Crystal	(2A4Mprm)_2_BiCl_5_·H_2_O	(2A4Mprm)_2_SbCl_5_·H_2_O	(2A4Mprm)SbBr_5_·H_2_O	(2A4Mprm)_4_Bi_2_Br_10_
Empirical formula	C_10_H_18_N_6_OBiCl_5_	C_10_H_18_N_6_OSbCl_5_	C_5_H_11_N_3_OSbBr_5_	C_20_H_32_N_12_Bi_2_Br_10_
Formula weight/g mol^−1^	624.53	537.30	650.47	1657.63
Temperature/K	100(2)	100(2)	100(2)	100(2)
Crystal system	Monoclinic	Monoclinic	Monoclinic	Triclinic
Space group	*P*2_1_/*m*	*P*2_1_/*m*	*P*2_1_/*c*	*P*1̄
*a*/Å	5.586(3)	5.626(3)	11.296(4)	9.775(4)
*b*/Å	18.595(6)	18.512(5)	8.333(3)	9.808(4)
*c*/Å	8.970(4)	8.957(4)	16.662(4)	11.435(5)
*α*	90.00	90.00	90.00	76.78(2)
*β*	90.66(2)	91.09(2)	108.10(2)	65.46(2)
*γ*	90.00	90.00	90.00	82.72(2)
*V*/Å^3^	931.7(7)	932.7(7)	1490.8(8)	970.2(7)
*Z*	2	2	4	1
Goodness-of-fit on *F*^2^	1.07	1.12	1.01	1.06
Flack parameter	—	—	—	—
CCDC number	2023493	2023495	2023492	2023494

In both structures, 2-amino-4-methylpyrimidine is protonated at the most basic nitrogen atom—N1 atom in Fig. 7a and b which allows the formation of cationic dimers (Fig. 7c) *via* highly directional N–H⋯N hydrogen bonds and a donor–acceptor distance of 3 Å. The dimers occupy the voids between the *trans*-[BiCl_5_]_∞_ and *pseudo-trans*-[SbCl_5_]_∞_ chains (Fig. 7g) and are engaged in a net of hydrogen bonds (HBs) connecting anionic and cationic substructures. Three types of HBs can be distinguished: (i) N–H⋯Cl4, 3.28–3.33 Å, ∼164°; (ii) N–H^+^⋯O1w with water molecule as the acceptor of HB, 2.88 Å, 165°; and (iii) O 1w–H⋯Cl4 with water molecule as the donor of HB, 3.10–3.26 Å, 155°–173°. Despite the same configuration of the intermolecular contacts, the difference in the coordination geometry of Sb^3+^ and Bi^3+^ atoms and the four HBs contacts accepted by Cl4 ligand – two from water molecules and two from the cationic dimers – contributes significantly to the observed elongation of Sb–Cl4 bond compared to the bismuth analog (Fig. 7h).

**Fig. 8:**
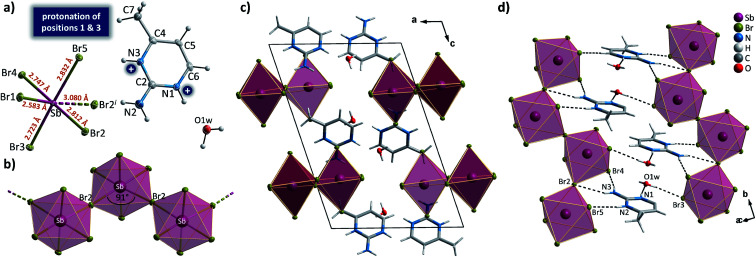
(a) The asymmetric part of the unit cell and the atom numbering scheme of (2A4Mprm)SbBr_5_·H_2_O at 100 K. Dashed lines correspond to symmetry-related atoms. Thermal ellipsoids are shown at the 50% probability level. (b) An anionic *cis*-[SbBr_5_]_∞_ chain. (c) Unit cell packing along the *b* axis. Intermolecular contacts are not shown for clarity. (d) A system of hydrogen bonds linking cationic and anionic substructures.

Crystals of bromoantimonate(iii) analog were identified as (2A4Mprm)SbBr_5_·H_2_O. The compound adopts the centrosymmetric *P*2_1_/*c* space group (Table 2); the asymmetric part of the unit cell is presented in Fig. 8a. The anionic substructure composes of SbBr_6_^3−^ octahedral units linked *via* Br2 ligands in *cis* position (∼90° Br2–Bi–Br2 angle) forming 1D *cis*-[SbCl_5_]_∞_ propagating in the *b*-axis direction (Fig. 8b). The cationic substructure is built of 2-amino-4-methylpyrimidine cations, 2A4Mprm^2+^, with positions 1 and 3 protonated (nitrogen atoms labeled N1 and N3 in Fig. 8a). To the best of our knowledge, this is the first example of diprotonation of 2A4Mprm, although such protonation is quite common in compounds of more symmetrical 2-aminopyrimidine.^55–57^ The lack of available HB acceptor in 2A4Mprm^2+^ prevents the formation of the cationic dimers typical for 2-aminopyrimidine derivatives. Instead, the cations are donors of a rich network of HBs with *cis*-[SbCl_5_]_∞_ chains (Fig. 8d). While N2 and N3 atoms connect the cations to the anionic chains *via* N–H⋯Br (3.32–3.39 Å, 138°–174°) and N–H^+^⋯Br bonds (3.32–3.39 Å, 138°–174°), respectively, N1 atoms along with water molecules form HB connections to the neighboring *cis*-[SbCl_5_]_∞_ chains *via* N1–H^+^⋯O1w (2.74 Å, 167°) and O1w–H⋯Br3 (3.41 Å, 143°) bonds.

**Fig. 9:**
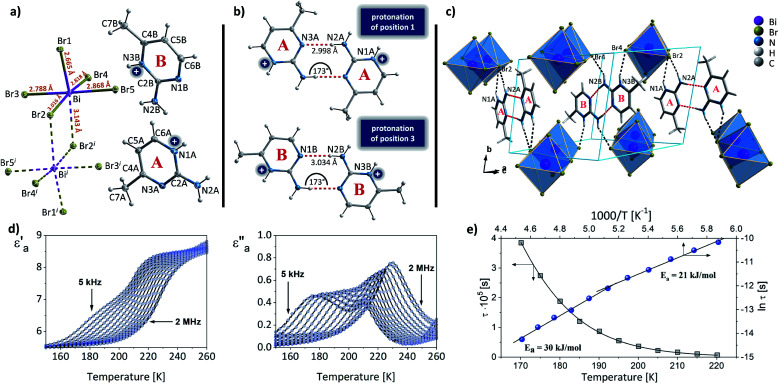
(a) The asymmetric part of the unit cell and the atom numbering scheme of (2A4Mprm)_4_Bi_2_Br_10_ at 100 K. Dashed lines correspond to symmetry-related atoms. Thermal ellipsoids are shown at the 50% probability level. (b) Two types of cationic dimers with geometrical parameters of their N–H⋯N hydrogen bonds. (c) The packing of the unit cell. (d) Temperature dependence of the real and imaginary parts of the complex electric permittivity obtained on heating (2A4Mprm)_4_Bi_2_Br_10_ crystal. (e) *τ vs. T* and ln *τ vs.* 1000/*T* plots along with the estimated activation energy values.

When incorporated into bromobismuthate(iii) network, 2A4Mprm gives (2A4Mprm)_4_Bi_2_Br_10_ complex. It crystallizes in *P*1̄ space group (Table 2) and composes of 0D Bi_2_Br_10_^4−^ units and two types of organic counterions (A and B in Fig. 9a). The cations differ in regards to their protonation sites: cation A is protonated at position 1 (N1A atom) which is the most basic site, cation B is protonated at position 3 (N3B atom), the less basic nitrogen atom in the ring. Similar protonation of 2-amino-4-methylpyrimidine has been found in only one structure described by Aakeröy *et al*.^58^ Both cations form dimers, with another molecule of its type (Fig. 9b) and are located either at the center of the unit cell (B⋯B dimer) or around the centers of symmetry at 0, ½, 0 (A⋯A dimer; Fig. 9c). A net of N–H⋯Br bonds stabilizes (3.32–3.40 Å, 133°–165°; Table S9†) their positions. Different configuration of HBs is a probable cause of the dynamics of the polar units observed in dielectric spectroscopy results. By using 5 kHz–2 MHz frequency range, very well-defined relaxation processes are observed along the crystallographic *a* axis: one in the high-frequency range right below room temperature, and the other in 155–200 K range driven by low frequencies (Fig. 9d). Both processes are polydispersive with *α* parameter values (from Cole–Cole equation) of 0.25–0.35 and 0.10–0.20, respectively, which indicates a contribution of at least a couple of relaxators to each of the processes (Table S12†). Nevertheless, only two activation energy values, 21 and 30 kJ mol^−1^, were obtained for these dynamics from the Arrhenius relation.

## Discussion

Three investigated organic cations – 2-mercaptopyrimidinium (2Sprm^+^), 2-aminopyrimidinium (2Aprm^+^), 2-amino-4-methylpyrimidinium (2A4Mprm^+^) – easily incorporate themselves into the halogenoantimonate(iii) and halogenobismuthate(iii) inorganic networks of R_2_MX_5_ stoichiometry. Overall, the cations show a strong tendency to form cationic dimers *via* N–H⋯S (2Sprm^+^ compounds) or N–H⋯N (2Aprm^+^ and 2A4Mprm^+^ compounds) hydrogen bonds. Although large in size, the dimers revealed their dynamic properties in a form of relaxation processes, observed in the dielectric spectroscopy results, with 20–60 kJ mol^−1^ activation energy values. The fact that these processes were observed mainly in bismuth-based systems—(2Sprm)_2_BiCl_5_, (2Aprm)_4_Bi_2_Cl_10_·H_2_O, and (2A4Mprm)_4_Bi_2_Br_10_—suggests that the size of voids occupied by organic cations and the strength of intermolecular interactions are major factors controlling the dynamics of polar units when the ac electric field is applied.

**Fig. 10:**
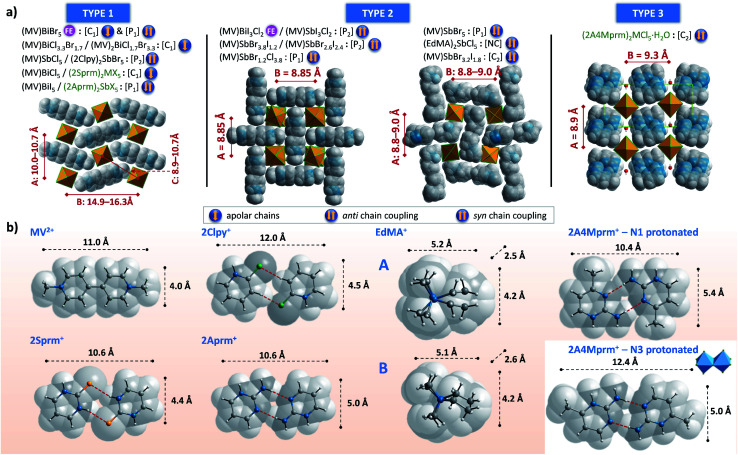
(a) Three types of crystal packing identified within R_2_MX_5_ stoichiometry with *trans*-[MX_5_]_∞_ chains. See text for symbols explanation. (b) Cationic units, along with their dimensions, leading to the formation of *trans*-[MX_5_]_∞_ chains. FE = ferroelectric.

**Fig. 11:**
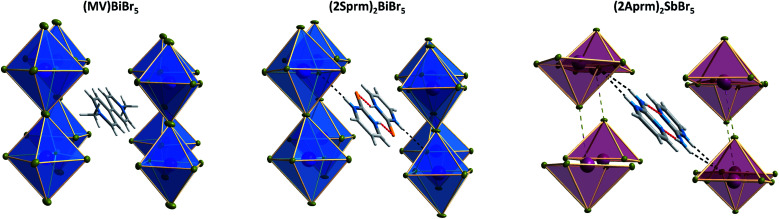
The position of cationic units in Type 1 crystal packing of (MV)BiBr_5_, (2Sprm)_2_BiBr_5_, and (2Aprm)_2_SbBr_5_ crystals.

As the results of the structural studies showed, only 2Sprm^+^ cation is accompanied in all four members of its family by *trans*-[MX_5_]_∞_ or *pseudo-trans*-[MX_5_]_∞_ chains. The other two cations – 2Aprm^+^ and 2A4Mprm^+^ – lead to the formation of *trans*-[MX_5_]_∞_ chains in two cases each: (2Aprm)_2_SbCl_5_ and (2Aprm)_2_SbBr_5_, and (2A4Mprm)_2_SbCl_5_·H_2_O and (2A4Mprm)_2_BiCl_5_·H_2_O, respectively. Nevertheless, these 8 compounds significantly increase the number of compounds with the desired *trans*-[MX_5_]_∞_ chains. In an attempt to understand the factors contributing to the formation of this specific anionic structure, a structural analysis of all compounds with the chains of *trans*-connected octahedra was performed. We found three types of crystal packing that are adopted by the compounds (Fig. 10a). While Types 1 and 2 are the most common ones and represent the packing of the two known ferroelectric compounds,^36,37^ (MV)BiBr_5_ and (MV)BiI_3_Cl_2_, respectively, Type 3 is adopted only by the two hydrates described in this article, (2A4Mprm)_2_SbCl_5_·H_2_O and (2A4Mprm)_2_BiCl_5_·H_2_O. The packings differ (i) in the relative alignment of the organic units within the crystal, (ii) in the distance between the adjacent anionic chains, and (iii) in the size of the voids occupied by cationic units (Types 1 or 2 *vs.* Type 3). In addition, each compound can be assigned to a category based on the symmetry of its structure and polarity of *trans*-[MX_5_]_∞_ chains and their coupling. Following Leblanc *et al.*,^48^ several categories can be distinguished: [*C*_1_] – centrosymmetric space group with apolar chains; [*C*_2_] – centrosymmetric space group with polar chains and *anti* chains coupling; [*P*_1_] – polar space group with *anti* chains coupling; [*P*_2_] – polar space group with *syn* chains coupling; and [NC] – noncentrosymmetric space group with *anti* chains coupling. Here, the [NC] category was added to the list after the identification of (EdMA)_2_SbCl_5_ compound (where EdMA^+^ is ethyldimethylammonium) crystallizing in noncentrosymmetric *P*2_1_2_1_2_1_ space group with *anti* chains coupling (Type 2 in Fig. 10a). Possible restrictions in the choice of organic cations were evaluated by scrutinizing the size of the organic units that accompany the inorganic *trans*-[MX_5_]_∞_ chains. The cations, or cationic dimeric forms, along with their dimensions are depicted in Fig. 10b. In general, the size of all of the cationic forms is in the ranges of 10.4–12 Å in length (for EDMA^+^ it is the length of two cations; right panel in Type 2 in Fig. 10a†) and 4.0–5.4 Å in width (a shorter in-plane axis). In the case of the most common crystal packings, Types 1 and 2, it was determined that while the crystal structures are substantially flexible to accommodate longer cations by increasing the distances between the adjacent chains, there is a limit in the allowed width of the units. The closest packing in these crystals is achieved by positioning the organic units in the voids at a 20°–45° angle relative to the direction of the chains (Fig. 11). Wider units would require the chains to expand by increasing their metal–halogen–metal distance. To some extent, this can be accomplished by stereochemical activation of ns^2^ lone electron pair of Sb^3+^ and Bi^3+^ ions allowing for the substantial deformation of octahedral units along the chain direction and leading to the formation of polar *trans*-[MX_5_]_∞_ or *pseudo-trans*-[MX_5_]_∞_ chains.^59^ When the expansion threshold is reached, either a new type of packing is formed, as in the case of (2A4Mprm)_2_SbCl_5_·H_2_O and (2A4Mprm)_2_BiCl_5_·H_2_O, or the arrangement with 0D inorganic units (M_2_X_10_^4−^) is preferred. The effort of the inorganic framework to accommodate a cationic unit is particularly well visualized in the family of 2Sprm^+^ (Fig. 1 and 2) where the cationic dimer fits well the voids in the systems with larger octahedral units (BiBr and BiCl) but causes weaker (SbBr) or stronger (SbCl) deformation of the systems characterized by shorter metal–ligand distances.

Of particular importance are interactions between cations and inorganic chains which might promote or disrupt the formation of *trans*-[MX_5_]_∞_ chains. In the case of MV^2+^ cation which gave a series of compounds with *trans*-[MX_5_]_∞_ chains,^36,37,48^ its 4 Å width seems to match well the voids in the structure and its lack of ability to form strong hydrogen bonds might not only ease its incorporation but also can ease the adjustment of its position when the polarization of the chains is switched in the two known ferroelectric cases. While an introduction of a stronger proton-donating group into an aromatic ring, NH in 2Sprm^+^, 2Clpy^+^, and 2Aprm^+^, helps in stabilizing the cations *via* highly directional (165°–170°) N–H⋯X hydrogen bonds of moderate strength, attaching the group closer to the center of the cationic unit might not necessarily provide better stabilization. For instance, the NH_2_ group in 2Aprm^+^ on the one hand plays a crucial role in the formation of the dimeric unit with the desired size (*via* N–H⋯N HBs), but, on the other hand, it introduces another proton that needs to find its acceptor. In (2Aprm)_2_SbCl_5_ and (2Aprm)_2_SbBr_5_, the proton is involved in the strained (141–147°) N–H⋯X contact. When changing the inorganic framework to larger chloro- and bromobismuthates(iii), even more strain would be expected. It is, therefore, plausible that this small structural feature prevents the observation of a complete 2Aprm^+^ family of compounds with *trans*-[MX_5_]_∞_ chains, as is observed for 2Sprm^+^. Instead, two compounds with Bi_2_X_10_^4−^ units are obtained: (2Aprm)_4_Bi_2_Cl_10_·H_2_O and (2Aprm)_4_Bi_2_Br_10_.

While general guidelines regarding a choice or design of appropriate cations can be extracted from this small database of compounds with *trans*-[MX_5_]_∞_ chains, there is no simple procedure for obtaining a polar, potentially ferroelectric, material. However, more control over the polarity of the chains might be introduced by using mixed halide systems, for instance, Cl/I. As it is well known,^36,37^ a combination of (MV)BiCl_5_ of [*C*_1_] category and (MV)BiI_5_ of [*P*_1_] category (Fig. 10a) leads to the formation of ferroelectric (MV)BiI_3_Cl_2_ of [*P*_2_] category. Not only remarkable Cl/I segregation is observed in the formed polar chains, but also the chains are arranged in *syn* coupling configuration and are aligned along the 4-fold polar axis of the tetragonal system. These factors give rise to exceptionally high values of spontaneous polarization that can be switched with the electric field by changing the position of bridging halogen atoms.^37^ The suggested displacive mechanism of this switching rises, however, a question whether the switching can be accomplished only in *trans*-[MX_5_]_∞_ chains with metal centers in complete octahedral geometry (most bismuth-based complexes) or also in *pseudo-trans*-[MX_5_]_∞_ chains with sixth distance much longer than a typical M–X distance (most antimonate-based complexes). More investigations are needed to provide guidance in this regard.

## Conclusions

In summary, the tendency of pyrimidines to form dimeric cationic units is particularly beneficial in the networks of halogenoantimonates(iii) and halogenobismuthates(iii) of R_2_MX_5_ stoichiometry. In the majority of the investigated cases, the dimers of 2-mercaptopyrimidinium, 2-aminopyrimidinium, and 2-amino-4-methylpyrimidinium are accompanied in crystals by highly desired, in terms of ferroelectric properties, *trans*-[MX_5_]_∞_ inorganic chains. Two compounds were found to adopt polar space groups and are therefore potentially ferroelectric. We attribute the observed abundance of structures with the anionic unit considered extremely rare to the void-matching size of the cations. More precisely, based on the analysis of all available structures with *trans*-[MX_5_]_∞_ chains, we found that the formation of the chains is favored for the organic units that are 10.0–12.0 Å in length and 4.0–5.4 Å in width. These dimensions seem optimum for the cations to fill the voids between the chains in the three identified types of crystal packing. Although intermolecular interactions can strongly affect the formation of the chains, no firm conclusion in this regard could be reached from our study, hence warranting further investigations. Nevertheless, we anticipate that the presented results will serve as the first step in establishing a pathway towards the preparation of the halogenoantimonates(iii) and halogenobismuthates(iii) hybrids with high spontaneous polarization values resulting from ferroelectric switching mechanism now encountered mainly in inorganic multiferroics.

## Conflicts of interest

There are no conflicts to declare.

## Supplementary Material

RA-011-D0RA10151F-s001

RA-011-D0RA10151F-s002
