# Supplementary material for: Towards ferroelectricity-inducing chains of halogenoantimonates(iii) and halogenobismuthates(iii)
Source: RSC Adv. 2021 May 13;11(29):17574–86. doi: 10.1039/d0ra10151f (PMC9033163; doi:10.1039/d0ra10151f)
Supplement: RA-011-D0RA10151F-s001 [file RA-011-D0RA10151F-s001.pdf]

Electronic Supplementary Material (ESI) for RSC Advances.
This journal is © The Royal Society of Chemistry 2021

Towards ferroelectricity-inducing chains of halogenoantimonates(III) and halogenobismuthates(III)

Magdalena Owczarek,[1,*] Przemysław Szklarz, Ryszard Jakubas

Faculty of Chemistry, University of Wroclaw, F. Joliot Curie 14, 50-383 Wroclaw, Poland.

[1]*Current address: Materials Physics and Applications Division, Center for Integrated Nanotechnologies, Los Alamos National Laboratory, Los Alamos, New Mexico 87545, USA*

magdalena@lanl.gov

SUPPORTING INFORMATION

Table of Contents

1. TGA & DTA results 2
2. Single-crystal X-ray diffraction 3
   a) 2-mercaptopyrimidinium family 3
   b) 2-aminopyrimidinium family 5
   c) 2-amino-4-methylpyrimidinium family 8
3. Dielectric spectroscopy 11
   a) $(2Sprm)_2BiCl_5$ 11
   b) $(2Aprm)_2SbCl_5$ 11
   c) $(2Aprm)_4Bi_2Cl_{10} \cdot 2H_2O$ 12
   d) $(2A4Mprm)_4Bi_2Br_{10}$ 12
4. References 13

1. TGA & DTA results

❖ 2-mercaptopyrimidinium family

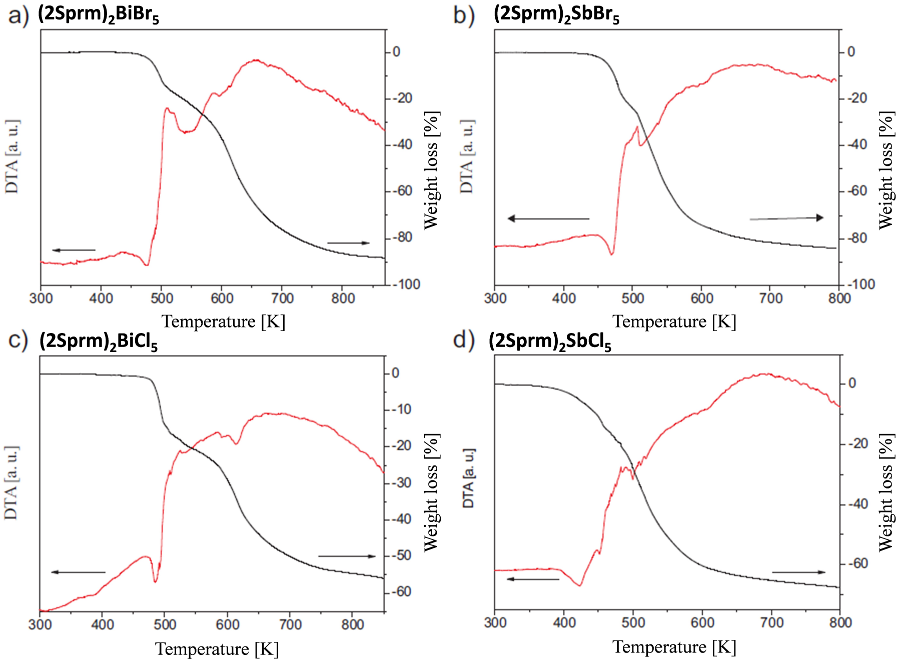

Figure S1. Thermogravimetric (TGA) and differential thermal (DTA) analyses scans obtained for a) **$(2Sprm)_2BiBr_5$** / m = 13.894 mg; b) **$(2Sprm)_2SbBr_5$** / m = 9.026 mg; c) **$(2Sprm)_2BiCl_5$** / m = 12.682 mg; d) **$(2Sprm)_2SbCl_5$** / m = 7.310 mg.

❖ 2-aminopyrimidinium family

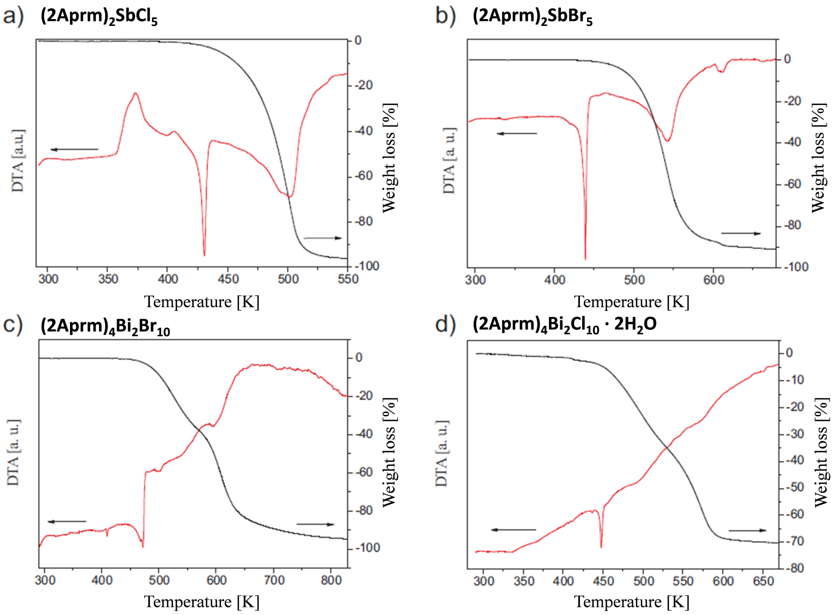

Figure S2. Thermogravimetric (TGA) and differential thermal (DTA) analyses scans obtained for a) **$(2Aprm)_2SbCl_5$** / m = 8.420 mg; b) **$(2Aprm)_2SbBr_5$** / m = 14.024 mg; c) **$(2Aprm)_4Bi_2Br_{10}$** / m = 9.788 mg; d) **$(2Aprm)_4Bi_2Cl_{10} \cdot H_2O$**/ m = 7.158 mg.

❖ 2-amino-4-methylpyrimidinium family

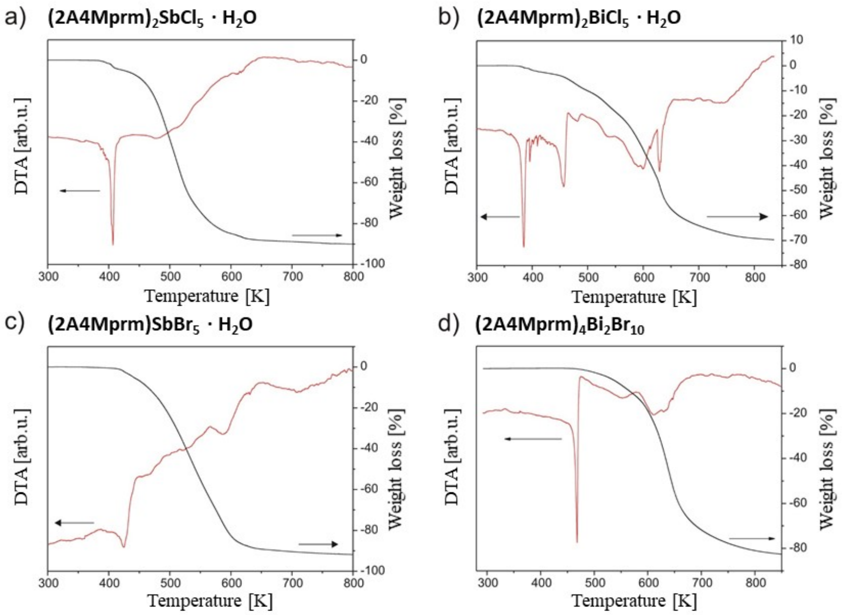

Figure S3. Thermogravimetric (TGA) and differential thermal (DTA) analyses scans obtained for a) **$(2A4Mprm)_2SbCl_5 \cdot H_2O$** / m = 7.238 mg; b) **$(2A4Mprm)_2BiCl_5 \cdot H_2O$** / m = 12.160 mg; c) **$(2A4Mprm)SbBr_5 \cdot H_2O$** / m = 8.982 mg; d) **$(2A4Mprm)_4Bi_2Br_{10}$** / m = 13.834 mg.

2. Single-crystal X-ray diffraction

a) 2-mercaptopyrimidinium family

The X-ray diffraction data of the **2Sprm** crystals were collected at 100K using Oxford Diffraction Xcalibur diffractometer with Onyx CCD detector and Mo Kα radiation. Data collection and reduction were carried out with CrysAlis CCD and CrysAlis PRO, respectively. Using Olex2,[1] crystal structures were solved by the Patterson method (SHELXS[2] program and refined with the ShelXL[2] with anisotropic thermal parameters for non-H atoms. The H atoms of the aromatic CH and NH groups were refined by means of a riding model with fixed C–H and N–H distances equal to 0.95 and 0.88 Å and U_{iso}(H) = 1.2U_{eq}(C or N). Crystallographic data for the structure reported have been deposited with the Cambridge Crystallographic Data Centre, CCDC Nos. 2023489–2023491. Copies of this information may be obtained free of charge via www.ccdc.cam.ac.uk/data_request/cif website.

Table S1. Crystal data, data collection and structure refinement parameters for **2Sprm** crystals.

	**$(2Sprm)_2SbCl_5$**	**$(2Sprm)_2BiCl_5$**	**$(2Sprm)_2BiBr_5$**
Empirical formula	$C_8H_{10}N_4S_2SbCl_5$	$C_8H_{10}N_4S_2BiCl_5$	$C_8H_{10}N_4S_2BiBr_5$
Formula weight / g mol^{-1}	525.32	612.55	834.85
Temperature / K	100(2)	100(2)	100(2)

Wavelength / Å	0.71073	0.71073	0.71073
Crystal system	Monoclinic	Monoclinic	Monoclinic
Space group	$P2_1/n$	$P2_1/n$	$P2_1/n$
a / Å	5.540(4)	5.000(4)	5.752(4)
b / Å	14.910(5)	15.082(5)	15.263(5)
c / Å	10.210(5)	10.360(4)	10.633(4)
a	90.00	90.00	90.00
β	104.08(2)	104.39(2)	104.77(2)
γ	90.00	90.00	90.00
V / Å^3	818.0(8)	832.4(7)	902.7(8)
Z	2	2	2
D_{calc} / Mg m^{-3}	2.133	2.444	3.072
μ / mm^{-1}	2.751	11.636	21.058
F(000)	508	572	752
Crystal size / mm^3	0.26 × 0.13 × 0.07	0.20 × 0.16 × 0.13	0.13 × 0.12 × 0.12
θ range / °	4.9–33.7	4.7–38.5	4.8–28.7
Ranges of h, k, l	$-7 \le h \le 22$<br>$-22 \le k \le 22$<br>$-9 \le l \le 13$	$-8 \le h \le 9$<br>$-26 \le k \le 17$<br>$-16 \le l \le 17$	$-7 \le h \le 7$<br>$-20 \le k \le 11$<br>$-14 \le l \le 13$
Absorption correction	analytical	analytical	analytical
Reflections collected/unique	5036/2715	16766/4549	5070/2247
R_{int}	0.0304	0.0252	0.0338
Data/restraints/parameters	2715/2/97	4549/2/96	2247/2/94
Goodness-of-fit on F^2	1.05	1.02	1.03
Final R indices [$I > 2\sigma(I)$]	$R_1 = 0.0293$<br>$wR_2 = 0.0505$	$R_1 = 0.0189$<br>$wR_2 = 0.0348$	$R_1 = 0.0266$<br>$wR_2 = 0.0536$
R indices (all data)	$R_1 = 0.0420$<br>$wR_2 = 0.0561$	$R_1 = 0.0286$<br>$wR_2 = 0.0373$	$R_1 = 0.0355$<br>$wR_2 = 0.0566$
$\Delta\rho_{max}/\Delta\rho_{min}$ / e Å^{-3}	0.72/–0.81	1.48/–1.45	1.92/–1.56
CCDC number	2023489	2023490	2023491

Table S2. Geometric parameters of anionic units of **2Sprm** crystals.

**$(2Sprm)_2SbCl_5$**					
Sb—Cl3^{1}	2.6165(10) Å	Cl2—Sb—Cl3^{1}	90.17(3)°	Cl1—Sb—Cl2	91.67(4)°
Sb—Cl3	2.6164(10) Å	Cl2^{1}—Sb—Cl3^{1}	89.83(3)°	Cl1^{1}—Sb—Cl2	88.33(4)°
Sb—Cl2^{1}	2.5808(13) Å	Cl2^{1}—Sb—Cl3	90.17(3)°	Cl1^{1}—Sb—Cl2^{1}	91.67(4)°
Sb—Cl2	2.5808(13) Å	Cl2—Sb—Cl2^{1}	180.0°	Cl1—Sb—Cl2^{1}	88.33(4)°
Sb—Cl1^{1}	2.306(2) Å	Cl1^{1}—Sb—Cl3	88.51(3)°	Cl1—Sb—Cl1^{1}	180.00(6)°
Sb—Cl1	2.306(2) Å	Cl1^{1}—Sb—Cl3^{1}	91.49(3)°	Cl1^{2}—Cl1—Sb	152.56(18)°
Cl3—Sb—Cl3^{1}	180.0°	Cl1—Sb—Cl3	91.49(3)°		
Cl2—Sb—Cl3	89.83(3)°	Cl1—Sb—Cl3^{1}	88.51(3)°		
Symmetry code: 1: $-x+1, -y+1, -z+1$; 2: $-x, -y+1, -z+1$					
**$(2Sprm)_2BiCl_5$**					
Bi—Cl3^{1}	2.6859(9) Å	Cl3—Bi—Cl1^{2}	90.717(11)°	Cl2^{1}—Bi—Cl1^{2}	86.94(3)°
Bi—Cl3	2.6859(9) Å	Cl3—Bi—Cl1	89.829(11)°	Cl2—Bi—Cl1	86.94(3)°
Bi—Cl2^{1}	2.6537(10) Å	Cl2—Bi—Cl3^{1}	89.93(2)°	Cl2^{1}—Bi—Cl1	93.06(3)°
Bi—Cl2	2.6538(10) Å	Cl2^{1}—Bi—Cl3^{1}	90.07(2)°	Cl2—Bi—Cl1^{2}	93.06(3)°
Bi—Cl1^{1}	2.750(2) Å	Cl2—Bi—Cl3	90.07(2)°	Cl1^{2}—Bi—Cl1	180.0°
Bi—Cl1	2.750(2) Å	Cl2^{1}—Bi—Cl3	89.93(2)°	Bi^{3}—Cl1—Bi	180.0°
Cl3—Bi—Cl3^{1}	180.0°	Cl2^{1}—Bi—Cl2	180.0°		
Symmetry code: 1: $-x+2, -y+1, -z+1$; 2: $x+1, y, z$; 3: $x-1, y, z$					

**(2Sprm)$_2$BiBr$_5$**					
Bi—Br2^1	2.8004(11) Å	Br2^1—Bi— Br3^1	90.22(2)°	Br3—Bi—Br1	89.488(12)°
Bi—Br2	2.8004(11) Å	Br2—Bi—Br3	90.22(2)°	Br3^1—Bi—Br1	90.511(12)°
Bi—Br3	2.8219(9) Å	Br2^1—Bi—Br3	89.78(2)°	Br3—Bi—Br1^2	90.512(12)°
Bi—Br3^1	2.8219(9) Å	Br2^1—Bi—Br1^2	86.24(3)°	Br3^1—Bi—Br1^2	89.489(12)°
Bi—Br1^2	2.876(2) Å	Br2—Bi—Br1	86.24(3)°	Br1^2—Bi—Br1	180.0°
Bi—Br1	2.876(2) Å	Br2—Bi—Br1^2	93.76(3)°	Bi^3—Br1—Bi	180.0°
Br2^1—Bi—Br2	180.0°	Br2^1—Bi—Br1	93.76(3)°		
Br2—Bi—Br3^1	89.78(2)°	Br3—Bi—Br3^1	180.0°		
Symmetry code: 1: –*x*+3, –*y*+1, –*z*+1; 2: *x*+1, *y*, *z*; 3: *x*–1, *y*, *z*					

Table S3. Hydrogen bonds parameters (Å, °) of **2Sprm** crystals.

D—H···*A*	*D*—H [Å]	*H*···*A* [Å]	*D*···*A* [Å]	*D*—H···*A* [°]
**(2Sprm)$_2$SbCl$_5$**				
N1—H1···S^1	0.86	2.43	3.274(2)	169
N3—H3···Cl3^2	0.89	2.32	3.225(3)	168
Symmetry codes: 1: –*x*+1, –*y*+1, –*z*+2; 2: –*x*+3/2, *y*–1/2, –*z*+3/2				
**(2Sprm)$_2$BiCl$_5$**				
N1—H1···S^1	0.87	2.44	3.2852(17)	164
N3—H3···Cl3^2	0.85	2.36	3.203(2)	168
Symmetry codes: 1: –*x*+1, –*y*+1/2, –*z*; 2: –*x*+2, *y*+1/2, –*z*				
**(2Sprm)$_2$BiBr$_5$**				
N1—H1···S^1	0.88(2)	2.46	3.292(4)	157
N3—H3···Br3^2	0.87(2)	2.51	3.368(5)	168
Symmetry codes: 1: –*x*+1, –*y*+1, –*z*; 2: *x*–3/2, –*y*+1/2, *z*–1/2				

b) 2-aminopyrimidinium family

The X-ray diffraction data of the **2Aprm** crystals were collected at 100K or 120K [**(2Aprm)$_4$Bi$_2$Cl$_{10}$ · 2H$_2$O**)] using Oxford Diffraction Xcalibur diffractometer with Onyx or Sapphire2 CCD detectors and Mo Kα radiation. Data collection and reduction were carried out with CrysAlis CCD and CrysAlis PRO, respectively. Using Olex2[1], crystal structures were solved by Patterson or direct methods [**(2Aprm)$_4$Bi$_2$Br$_{10}$)**] (SHELXS program[2] and refined with the ShelXL[2] with anisotropic thermal parameters for non-H atoms. The H atoms of the aromatic groups—CH, NH—and NH$_2$ group were refined by means of a riding model with fixed C–H and N–H distances equal to 0.95 and 0.88 Å and U_{iso}(H) = 1.2U_{eq}(C or N). Water H atoms in **(2Aprm)$_4$Bi$_2$Br$_{10}$) · H$_2$O** were refined with a rigid model with O–H and H···H distances equal to 0.88 Å and 1.44 Å, respectively, and U_{iso}(H) = 1.5U_{eq}(O). Crystallographic data for the structure reported have been deposited with the Cambridge Crystallographic Data Centre, CCDC Nos. 2025386–2025389. Copies of this information may be obtained free of charge via www.ccdc.cam.ac.uk/data_request/cif website.

Table S4. Crystal data, data collection and structure refinement parameters for **2Aprm** crystals.

	**$(2Aprm)_2SbCl_5$**	**$(2Aprm)_2SbBr_5$**	**$(2Aprm)_4Bi_2Cl_{10} \cdot 2H_2O$**	**$(2Aprm)_4Bi_2Br_{10}$**
Empirical formula	$C_8H_{12}N_6SbCl_5$	$C_8H_{12}N_6SbBr_5$	$C_{16}H_{28}N_{12}O_2Bi_2Cl_{10}$	$C_8H_{12}N_6BiBr_5$
Formula weight / g mol^{-1}	491.06	713.54	1192.96	800.77
Temperature / K	100(2)	100(2)	120(2)	100(2)
Wavelength / Å	0.71073	0.71073	0.71073	0.71073
Crystal system	Monoclinic	Monoclinic	Monoclinic	Triclinic
Space group	$P2_1$	$P2_1$	$C2/c$	$P\bar{1}$
a / Å	5.713(3)	5.946(3)	19.226(4)	7.873(3)
b / Å	14.125(5)	14.401(4)	13.018(4)	11.404(4)
c / Å	10.254(4)	10.621(4)	14.193(4)	11.618(4)
a	90.00	90.00	90.00	115.80(5)
β	105.65(2)	105.31(2)	100.74(2)	96.30(4)
γ	90.00	90.00	90.00	98.86(4)
V / Å^3	796.8(6)	877.2(6)	3490.1(16)	909.4(7)
Z	2	2	4	2
D_{calc} / Mg m^{-3}	2.047	2.701	2.270	2.924
μ / mm^{-1}	2.566	12.959	10.875	20.676
F(000)	476	656	2240	720
Crystal size / mm^3	0.22 × 0.18 × 0.12	0.16 × 0.15 × 0.10	0.17 × 0.08 × 0.06	0.31 × 0.13 × 0.04
θ range / °	2.9–28.3	4.7–28.7	4.7–33.1	3.0–36.7
Ranges of h, k, l	$-7 \le h \le 7$<br>$-18 \le k \le 18$<br>$-13 \le l \le 13$	$-8 \le h \le 8$<br>$-15 \le k \le 19$<br>$-14 \le l \le 13$	$-29 \le h \le 14$<br>$-16 \le k \le 19$<br>$-21 \le l \le 21$	$-8 \le h \le 11$<br>$-14 \le k \le 14$<br>$-19 \le l \le 14$
Absorption correction	analytical	analytical	analytical	analytical
Reflections collected/unique	11515/3899	7214/3618	13638/6456	9692/5132
R_{int}	0.0205	0.0218	0.0344	0.048
Data/restraints/parameters	3899/1/182	3618/1/181	6456/3/196	5132/0/181
Goodness-of-fit on F^2	1.09	1.10	0.97	1.04
Final R indices [$I > 2\sigma(I)$]	$R_1 = 0.0184$<br>$wR_2 = 0.0445$	$R_1 = 0.0226$<br>$wR_2 = 0.0487$	$R_1 = 0.0301$<br>$wR_2 = 0.0414$	$R_1 = 0.0546$<br>$wR_2 = 0.1377$
R indices (all data)	$R_1 = 0.0188$<br>$wR_2 = 0.0447$	$R_1 = 0.0250$<br>$wR_2 = 0.0498$	$R_1 = 0.0498$<br>$wR_2 = 0.0463$	$R_1 = 0.0648$<br>$wR_2 = 0.1499$
$\Delta\rho_{max}/\Delta\rho_{min}$ / e Å^{-3}	0.80/–0.30	1.06/–0.52	1.66/–1.05	2.72/–2.76
Flack parameter	0.397(14)	0.446(13)	-	-
CCDC number	2025386	2025387	2025388	2025389

Table S5. Geometric parameters of anionic units of **2Aprm** crystals.

**$(2Aprm)_2SbCl_5$**					
Sb—Cl4	2.6536(12) Å	Cl3—Sb—Cl4	89.88(3)°	Cl5—Sb—Cl4	91.32(3)°
Sb—Cl3	2.5834(12) Å	Cl3—Sb—Cl2	87.80(3)°	Cl1—Sb—Cl1	86.47(3)°
Sb—Cl2	2.6119(12) Å	Cl3—Sb—Cl5	174.07(3)°	Cl1—Sb—Cl3	89.19(3)°
Sb—Cl5	2.6315(12) Å	Cl2—Sb—Cl4	174.97(3)	Cl1—Sb—Cl2	89.04(3)°
Sb—Cl1	2.3905(14) Å	Cl2—Sb—Cl5	90.54(3)°	Cl1—Sb—Cl5	85.08(3)°
**$(2Aprm)_2SbBr_5$**					
Sb—Br2	2.7662(14) Å	Br2—Sb—Br3	90.19(3)°	Br5—Sb—Br4	89.61(3)°
Sb—Br3	2.7796(13) Å	Br2—Sb—Br4	178.70(3)°	Br1—Sb—Br2	90.46(3)°
Sb—Br5	2.7599(13) Å	Br3—Sb—Br4	90.84(4)°	Br1—Sb—Br3	87.29(3)°
Sb—Br4	2.8021(14) Å	Br5—Sb—Br2	89.32(4)°	Br1—Sb—Br5	90.86(3)°

Sb—Br1	2.5594(15) Å	Br5—Sb—Br3	178.09(3)°	Br1—Sb—Br4	88.81(3)°
**$(2Aprm)_4Bi_2Cl_{10} \cdot 2H_2O$**					
Bi—Cl4	2.6096(11) Å	Cl4—Bi—Cl5[1]	91.76(3)°	Cl1—Bi—Cl5	92.70(3)°
Bi—Cl3	2.5171(9) Å	Cl4—Bi—Cl2	175.36(3)°	Cl1—Bi—Cl2	90.31(3)°
Bi—Cl1	2.6733(11) Å	Cl3—Bi—Cl4	88.64(3)°	Cl5[1]—Bi—Cl5	82.00(3)°
Bi—Cl5[1]	2.7800(12) Å	Cl3—Bi—Cl1	94.03(3)°	Cl5[1]—Bi—Cl2	89.15(3)°
Bi—Cl5	3.0260(10) Å	Cl3—Bi—Cl5	173.25(3)°	Cl2—Bi—Cl5	92.73(3)°
Bi—Cl2	2.7847(12) Å	Cl3—Bi—Cl5[1]	91.26(3)°	Bi[1]—Cl5—Bi	98.00(3)°
Cl4—Bi—Cl1	89.21(3)°	Cl3—Bi—Cl2	86.79(3)°		
Cl4—Bi—Cl5	91.90(3)°	Cl1—Bi—Cl5[1]	174.62(2)°		
Symmetry code: 1: –*x*+1/2, –*y*+1/2, –*z*+1					
**$(2Aprm)_4Bi_2Br_{10}$**					
Bi—Br5	2.8247(16) Å	Br5—Bi—Br4	91.52(4)°	Br1—Bi—Br2	90.44(5)°
Bi—Br3	2.9362(17) Å	Br3—Bi—Br3[1]	85.68(5)°	Br2—Bi—Br5	87.25(4)°
Bi —Br3[1]	3.090(3) Å	Br3—Bi—Br4	88.58(4)°	Br2—Bi—Br3	92.62(4)°
Bi —Br4	2.9644(14) Å	Br4—Bi—Br3[1]	89.10(4)°	Br2—Bi—Br3[1]	90.43(5)°
Bi —Br1	2.724(2) Å	Br1—Bi—Br5	95.08(6)°	Br2—Bi—Br4	178.67(3)°
Bi —Br2	2.7333(14) Å	Br1—Bi—Br3[1]	173.77(3)°	Bi—Br3—Bi[1]	94.32(5)°
Br5—Bi—Br3	176.79(3)°	Br1—Bi—Br3	88.12(6)°		
Br5—Bi—Br3[1]	91.12(5)°	Br1—Bi—Br4	90.16(5)°		
Symmetry code: 1: –*x*+1, –*y*, –*z*+1					

Table S6. Hydrogen bonds parameters (Å, °) of **2Aprm** crystals.

D—H···*A*	*D*—H [Å]	H···*A* [Å]	*D*···*A* [Å]	*D*—H···*A* [°]
**$(2Aprm)_2SbCl_5$**				
N2B—H2BA···N3A	0.88	2.09	2.965(4)	172
N2A—H2AB···N3B	0.88	2.10	2.975(4)	172
N2B—H2BB···Cl2[1]	0.88	2.91	3.635(3)	141
N1A—H1A···Cl4[2]	0.88	2.30	3.154(3)	163
N2A—H2AB···Cl4[2]	0.88	2.75	3.496(3)	143
N1B—H1B···Cl2[1]	0.88	2.34	3.197(3)	164
Symmetry codes: 1: –*x*+1, –*y*+1/2, –*z*+1; 2: –*x*, *y*–1/2, –*z*+1				
**$(2Aprm)_2SbBr_5$**				
N2B—H2BA···N3A	0.88	2.10	2.975(9)	174
N2A—H2AB···N3B	0.88	2.10	2.981(9)	176
N2B—H2BB···Br4[2]	0.88	2.84	3.602(7)	147
N1A—H1A···Br2[1]	0.88	2.50	3.354(6)	164
N2A—H2AA···Br2[1]	0.88	2.96	3.705(7)	144
N1B—H1B···Br4[2]	0.88	2.46	3.317(3)	164
Symmetry codes: 1: –*x*+1, –*y*+1/2, –*z*; 2: –*x*+2, *y*+1/2, –*z*				
**$(2Aprm)_4Bi_2Cl_{10} \cdot 2H_2O$**				
N1B—H1B···O1W[1]	0.88	1.79	2.663(4)	173
N2B—H2BB···N3A	0.88	2.05	2.934(4)	178
N2A—H2AA···N3B	0.88	2.24	3.114(4)	172
N1A—H1A···Cl1	0.88	2.37	3.199(3)	158
N2B—H2BA···Cl2[1]	0.88	2.42	3.245(3)	157
N2A—H2AB···Cl1	0.88	2.90	3.619(3)	140
O1W—H1WA···Cl5[2]	0.855(17)	2.77(3)	3.480(3)	141(4)
O1W—H1WA···Cl2	0.866(18)	2.57(2)	3.395(4)	159(4)
Symmetry codes: 1: –*x*, –*y*+1, –*z*+1; 2: *x*, –*y*+1, *z*–1/2				
**$(2Aprm)_4Bi_2Br_{10}$**				

N2A—H2AA···N3A^{2}	0.88	2.08	2.949(10)	169
N1A—H1A···Br5^{1}	0.88	2.49	3.241(9)	144
N2A—H2AB···Br3	0.88	2.92	3.646(8)	141
N2B—H2BA···Br3	0.88	2.69	3.487(10)	152
N2B—H2BB···Br5^{1}	0.88	2.60	3.440(11)	161
N1B—H1B···Br4^{3}	0.88	2.48	3.291(9)	154
Symmetry codes:1: *x*+1, *y*, *z*; 2: –*x*+2, –*y*+1, –*z*+1; 3: –*x*+1, –*y*, –*z*+1				

c) 2-amino-4-methylpyrimidinium family

The X-ray diffraction data for **2A4Mprm** crystals were collected at 100K using Oxford Diffraction Xcalibur diffractometer with Onyx or Sapphire2 CCD detectors and Mo Kα radiation. Data collection and reduction were carried out with CrysAlis CCD and CrysAlis PRO, respectively. Using Olex2[1], crystal structures of **(2A4Mprm)$_2$SbCl$_5$ · H$_2$O, (2A4Mprm)$_2$BiCl$_5$ · H$_2$O,** and **(2A4Mprm)$_4$Bi$_2$Br$_{10}$** were solved by Patterson method (SHELXS program[2]); crystal structure of **(2A4Mprm)SbBr$_5$ · H$_2$O** was solved by direct methods (SHELXS program[2]). The structures were refined with the ShelXL[2] with anisotropic thermal parameters for non-H atoms. Water H atoms were refined with a rigid model with O–H and H···H distances equal to 0.88 Å and 1.44 Å, respectively, and U_{iso}(H) = 1.5U_{eq}(O). The H atoms of the aromatic CH and NH groups, and NH$_2$ group on the second position of **2A4Mprm**, were refined by means of a riding model with fixed C–H and N–H distances equal to 0.95 and 0.88 Å and U_{iso}(H) = 1.2U_{eq}(C or N). The CH$_3$ groups were refined as rotating groups with a fixed C–H distance of 0.98 Å and U_{iso}(H) = 1.5U_{eq}(C). Crystallographic data for the structure reported have been deposited with the Cambridge Crystallographic Data Centre, CCDC Nos. 2023492–2023495. Copies of this information may be obtained free of charge via www.ccdc.cam.ac.uk/data_request/cif website.

Table S7. Crystal data, data collection and structure refinement parameters of **2A4Mprm** crystals.

	**(2A4Mprm)$_2$SbCl$_5$ · H$_2$O**	**(2A4Mprm)$_2$BiCl$_5$ · H$_2$O**	**(2A4Mprm)SbBr$_5$ · H$_2$O**	**(2A4Mprm)$_4$Bi$_2$Br$_{10}$**
Empirical formula	$C_{10}H_{18}N_6OSbCl_5$	$C_{10}H_{18}N_6OBiCl_5$	$C_5H_{11}N_3OSbBr_5$	$C_{20}H_{32}N_{12}Bi_2Br_{10}$
Formula weight / g mol^{-1}	537.30	624.53	650.47	1657.63
Temperature / K	100(2)	100(2)	100(2)	100(2)
Wavelength / Å	0.71073	0.71073	0.71073	0.71073
Crystal system	Monoclinic	Monoclinic	Monoclinic	Triclinic
Space group	$P2_1/m$	$P2_1/m$	$P2_1/c$	$P\bar{1}$
a / Å	5.626(3)	5.586(3)	11.296(4)	9.775(4)
b / Å	18.512(5)	18.595(6)	8.333(3)	9.808(4)
c / Å	8.957(4)	8.970(4)	16.662(4)	11.435(5)
a	90.00	90.00	90.00	76.78(2)
β	91.09(2)	90.66(2)	108.10(2)	65.46(2)
γ	90.00	90.00	90.00	82.72(2)
V / Å^3	932.7(7)	931.7(7)	1490.8(8)	970.2(7)
Z	2	2	4	1
D_{calc} / Mg m^{-3}	1.913	2.226	2.898	2.837
μ / mm^{-1}	2.206	10.19	15.234	19.386

$F(000)$	528	592	1184	752
Crystal size / mm^3	0.33 × 0.25 × 0.19	0.38 × 0.24 × 0.15	0.22 × 0.13 × 0.06	0.19 × 0.16 × 0.12
θ range / º	3.2–36.8	3.2–28.7	4.9–31.5	2.9–27.1
Ranges of h, k, l	$-8 \le h \le 9$ $-30 \le k \le 30$ $-14 \le l \le 14$	$-7 \le h \le 7$ $-23 \le k \le 25$ $-10 \le l \le 11$	$-16 \le h \le 16$ $-9 \le k \le 12$ $-24 \le l \le 24$	$-12 \le h \le 12$ $-12 \le k \le 12$ $-14 \le l \le 14$
Absorption correction	analytical	analytical	analytical	analytical
Reflections collected/unique	16596/4576	6440/2332	17564/4942	9141/4262
R_{int}	0.020	0.030	0.055	0.020
Data/restraints/parameters	4576/3/117	2332/3/117	4942/3/143	4262/0/201
Goodness-of-fit on F^2	1.12	1.07	1.01	1.06
Final R indices [$I > 2\sigma(I)$]	$R_1 = 0.0180$ $wR_2 = 0.0438$	$R_1 = 0.0226$ $wR_2 = 0.0557$	$R_1 = 0.0354$ $wR_2 = 0.0577$	$R_1 = 0.0207$ $wR_2 = 0.0505$
R indices (all data)	$R_1 = 0.0196$ $wR_2 = 0.0443$	$R_1 = 0.0233$ $wR_2 = 0.0561$	$R_1 = 0.0623$ $wR_2 = 0.0670$	$R_1 = 0.0232$ $wR_2 = 0.0515$
$\Delta\rho_{max}/\Delta\rho_{min}$ / e Å^{-3}	1.15/–0.35	1.75/–1.26	1.33/–1.34	1.13/–0.86
CCDC number	2023495	2023493	2023492	2023494

Table S8. Geometric parameters of anionic units of **2A4Mprm** crystals.

**$(2A4Mprm)_2SbCl_5 \cdot H_2O$**					
Sb—Cl1^1	2.5910(7) Å	Cl1—Sb—Cl1^1	171.939(12)°	Cl2—Sb—Cl4	175.840(11)°
Sb—Cl1	2.5910(7) Å	Cl1^1—Sb—Cl4	92.847(6)°	Cl3—Sb—Cl1	87.138(6)°
Sb—Cl4	3.0157(14) Å	Cl1—Sb—Cl4	92.847(6)°	Cl3—Sb—Cl1^1	87.138(6)°
Sb—Cl2	2.4472(11) Å	Cl2—Sb—Cl1^1	87.368(6)°	Cl3—Sb—Cl4	90.24(2)°
Sb—Cl3	2.3971(13) Å	Cl2—Sb—Cl1	87.367(6)°	Cl3—Sb—Cl2	93.92(2)°
Symmetry code: 1: x, –y+1/2, z					
**$(2A4Mprm)_2BiCl_5 \cdot H_2O$**					
Bi—Cl1^1	2.6602(12) Å	Cl2—Bi—Cl1^1	87.773(16)°	Cl1^1—Bi—Cl3^2	91.452(16)°
Bi—Cl1	2.6601(12) Å	Cl2—Bi—Cl3	94.96(4)°	Cl3—Bi—Cl4	92.09(4)°
Bi—Cl4	2.8840(17) Å	Cl2—Bi—Cl3^2	84.38(4)°	Cl3—Bi—Cl1^1	88.520(16)°
Bi—Cl2	2.5732(15) Å	Cl4—Bi—Cl3^2	88.56(4)°	Cl3—Bi—Cl1	88.520(16)°
Bi—Cl3	2.6322(19) Å	Cl1^1—Bi—Cl4	92.416(17)°	Cl3—Bi—Cl3^2	179.34(5)°
Bi—Cl3^2	2.954(2) Å	Cl1—Bi—Cl4	92.417(17)°	Bi—Cl3—Bi^3	179.34(5)°
Cl2—Bi—Cl4	172.95(3)°	Cl1^1—Bi—Cl1	174.42(3)°		
Cl2—Bi—Cl1	87.773(16)°	Cl1—Bi—Cl3^2	91.453(16)°		
Symmetry codes: 1: x, $-y+1/2$, z; 2: $x+1$, y, z; 3: $x-1$, y, z					
**$(2A4Mprm)SbBr_5 \cdot H_2O$**					
Sb—Br2	2.8115(9) Å	Br2—Sb—Br5	89.67(2)°	Br3—Sb—Br5	177.421(16)°
Sb—Br4	2.7471(8) Å	Br4—Sb—Br2	179.820(19)°	Br5—Sb—Br2^1	83.58(2)°
Sb—Br2^1	3.0799(9) Å	Br4—Sb—Br2^1	89.07(3)°	Br1—Sb—Br2^1	174.377(16)°
Sb—Br3	2.7229(8) Å	Br4—Sb—Br5	90.20(2)°	Br1—Sb—Br2	90.00(3)°
Sb—Br5	2.8317(9) Å	Br3—Sb—Br2^1	93.91(2)°	Br1—Sb—Br4	90.13(3)°
Sb—Br1	2.5829(8) Å	Br3—Sb—Br2	89.78(2)°	Br1—Sb—Br3	91.66(2)°
Br2—Sb—Br2^1	90.79(3)°	Br3—Sb—Br4	90.34(2)°	Br1—Sb—Br5	90.86(2)°
Symmetry code: 1: $-x$, $y+1/2$, $-z+1/2$					
**$(2A4Mprm)_4Bi_2Br_{10}$**					
Bi1—Br5	2.8676(12) Å	Br4—Bi1—Br5	90.14(2)°	Br1—Bi1—Br4	96.88(3)°
Bi1—Br4	2.8182(12) Å	Br4—Bi1—Br2	172.732(12)°	Br1—Bi1—Br3	90.50(3)°
Bi1—Br3	2.7878(12) Å	Br4—Bi1—Br2^1	89.10(3)°	Br1—Bi1—Br2^1	171.870(12)°
Bi1—Br1	2.6651(13) Å	Br3—Bi1—Br5	178.580(12)°	Br1—Bi1—Br2	90.37(3)°
Bi1—Br2^1	3.1432(15) Å	Br3—Bi1—Br4	89.19(2)°	Br2—Bi1—Br2^1	83.64(3)°

Bi1—Br2	3.0155(14) Å	Br3—Bi1—Br2	90.29(2)°	Bi1—Br2—Bi1[1]	96.36(3)°
Br5—Bi1—Br2[1]	97.18(3)°	Br3—Bi1—Br2[1]	84.06(3)°		
Br5—Bi1—Br2	90.53(2)°	Br1—Bi1—Br5	88.34(3)°		
Symmetry code: 1: –*x*, –*y*, –*z*+1					

Table S9. Hydrogen bonds parameters (Å, °) of **2A4Mprm** crystals.

D—H···*A*	*D*—H [Å]	*H*···*A* [Å]	*D*···*A* [Å]	*D*—H···*A* [°]
**$(2A4Mprm)_2SbCl_5 \cdot H_2O$**				
N1—H1···O1W	0.88	2.01	2.8736(12)	165
N2—H2A···N3[1]	0.88	2.14	3.0197(15)	178
N2—H2B···Cl4	0.88	2.42	3.2750(11)	165
O1W—H1WA···Cl4	0.870(15)	2.289(15)	3.0971(17)	154.6(19)
O1W—H1WB···Cl4[2]	0.861(15)	2.408(15)	3.2644(19)	172.8(19)
Symmetry codes: 1: –*x*+1, –*y*+1, –*z*; 2: *x*–1, *y*, *z*				
**$(2A4Mprm)_2BiCl_5 \cdot H_2O$**				
N1—H1···O1W	0.88	2.02	2.882(3)	165
N2—H2A···N3[1]	0.88	2.13	3.008(4)	179
N2—H2B···Cl4	0.88	2.48	3.333(3)	163
O1W—H1WB···Cl4	0.867(19)	2.31(2)	3.125(4)	157(4)
O1W—H1WA···Cl4[2]	0.870(19)	2.40(2)	3.263(4)	171(4)
Symmetry codes: 1: –*x*+1, –*y*+1, –*z*; 2: *x*–1, *y*, *z*				
**$(2A4Mprm)SbBr_5 \cdot H_2O$**				
N3—H3···Br5	0.88	2.44	3.321(4)	175
N2—H2A···Br4[1]	0.88	2.51	3.348(4)	160
N2—H2B···Br2	0.88	2.68	3.384(4)	138
N1—H1···O1W	0.86	1.89	2.739(5)	167
O1W—H1WA···Br3[2]	0.900(18)	2.66(3)	3.412(3)	142(4)
Symmetry codes: 1: –*x*, *y*–1/2, –*z*+1/2; 2: *x*+1, –*y*+3/2, *z*+1/2				
**$(2A4Mprm)_4Bi_2Br_{10}$**				
N2A—H2AA···N3A[3]	0.88	2.12	2.997(5)	173
N2B—H2BAA···N1B[1]	0.88	2.16	3.036(5)	173
N1—H1···Br2[4]	0.88	2.91	3.669(4)	146
N3B—H3B···Br4	0.88	2.48	3.315(3)	159
N2B—H2BB···Br4[2]	0.88	2.75	3.409(4)	133
N2A—H2AB···Br2[4]	0.88	2.50	3.354(4)	165
Symmetry codes:1: –*x*+1, –*y*+1, –*z*+1; 2: –*x*+1, –*y*, –*z*+1; 3: –*x*, –*y*+1, –*z*+2; 4: –*x*, –*y*+1, –*z*+1				

3. Dielectric spectroscopy

a) $(2Sprm)_2BiCl_5$

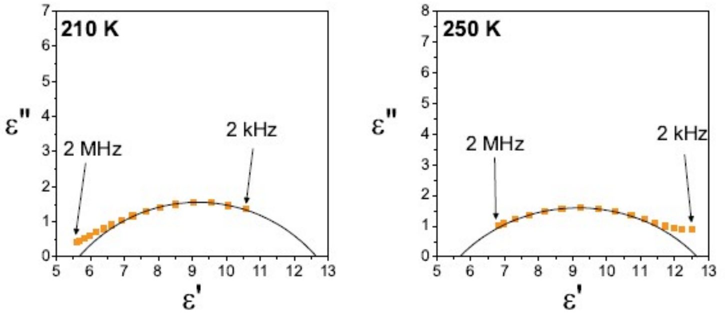

Figure S4. Cole-Cole plots of ε” vs ε’ at selected temperatures obtained for $\mathbf{(2Sprm)_2BiCl_5}$ crystal.

Table S10. Values of the parameters of Cole-Cole relation at selected temperatures for $\mathbf{(2Sprm)_2BiCl_5}$ crystal.

T [K]	ε_0	ε_∞	α	τ [s]
**270**	12.50	5.56	0.40	$1.52 \cdot 10^{-7}$
**260**	12.70	5.69	0.43	$4.40 \cdot 10^{-7}$
**250**	12.67	5.67	0.46	$1.01 \cdot 10^{-7}$
**235**	12.67	5.66	0.47	$3.19 \cdot 10^{-6}$
**220**	12.67	5.66	0.48	$8.77 \cdot 10^{-6}$
**205**	12.67	5.67	0.47	$3.16 \cdot 10^{-5}$

b) $(2Aprm)_2SbCl_5$

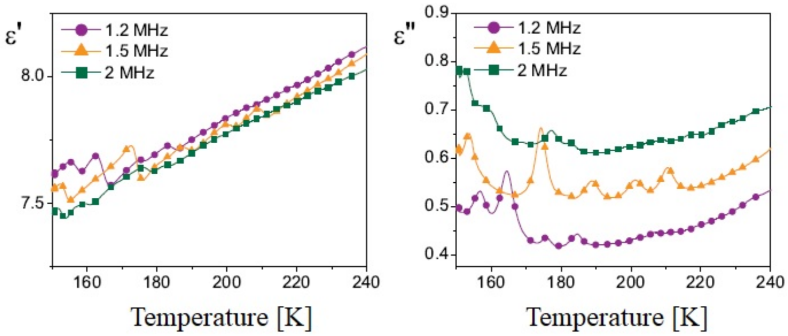

Figure S5. Temperature-dependent real and imaginary parts of electric permittivity obtained along [101] direction on cooling $\mathbf{(2Aprm)_2SbCl_5}$ crystal.

c) $(2Aprm)_4Bi_2Cl_{10} \cdot 2H_2O$

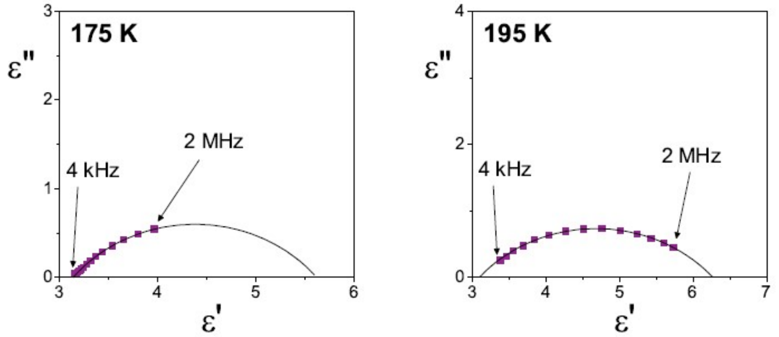

Figure S6. Cole-Cole plots of ε” vs ε’ at selected temperatures obtained for **$(2Aprm)_4Bi_2Cl_{10} \cdot 2H_2O$** crystal.

Table S11. Values of the parameters of Cole-Cole relation at selected temperatures for **$(2Aprm)_4Bi_2Cl_{10} \cdot 2H_2O$** crystal.

T [K]	ε_0	ε_∞	α	τ [s]
**195**	6.27	3.10	0.45	$3.05 \cdot 10^{-6}$
**190**	6.16	3.11	0.45	$7.51 \cdot 10^{-6}$
**185**	6.01	3.14	0.43	$1.70 \cdot 10^{-5}$
**180**	5.89	3.14	0.43	$3.72 \cdot 10^{-5}$
**175**	5.62	3.14	0.43	$9.84 \cdot 10^{-5}$
**170**	5.22	3.14	0.42	$2.88 \cdot 10^{-4}$

d) $(2A4Mprm)_4Bi_2Br_{10}$

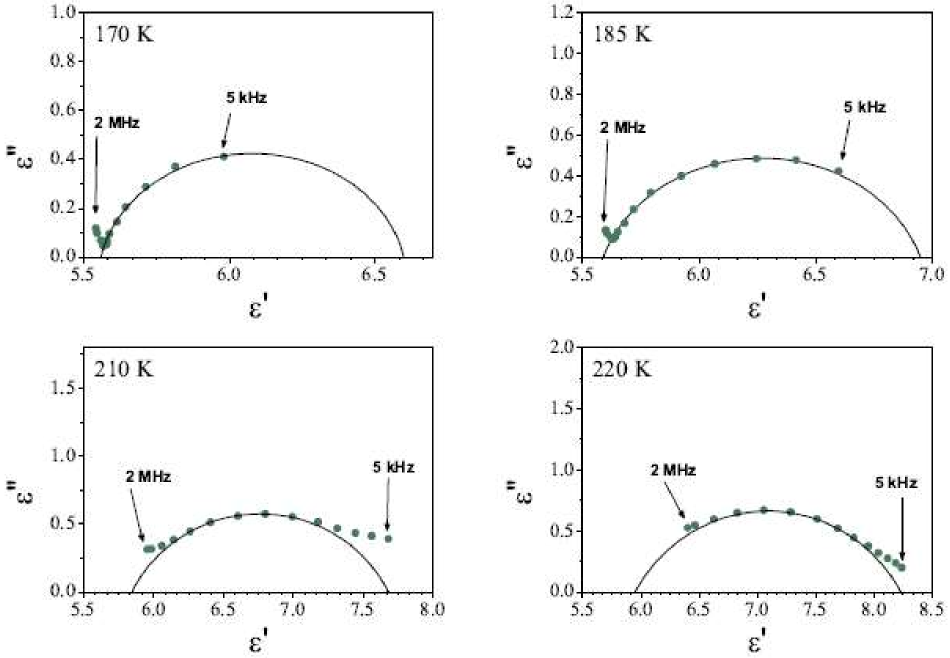

Figure S7. Cole-Cole plots of ε” vs ε’ at selected temperatures obtained for **$(2A4Mprm)_4Bi_2Br_{10}$** crystal.

Table S12. Values of the parameters of Cole-Cole relation at selected temperatures for **$(2A4Mprm)_4Bi_2Br_{10}$** crystal.

T [K]	ε_0	ε_∞	α	τ [s]
**170**	6.60	5.56	0.13	$3.85 \cdot 10^{-5}$
**180**	6.87	5.59	0.19	$1.88 \cdot 10^{-5}$
**190**	7.10	5.60	0.25	$8.62 \cdot 10^{-6}$
**200**	7.30	5.68	0.28	$3.62 \cdot 10^{-6}$
**210**	7.75	5.82	0.32	$1.61 \cdot 10^{-6}$
**220**	8.23	5.95	0.33	$6.46 \cdot 10^{-7}$

4. References

1. O. V. Dolomanov, L. J. Bourhis, R. J. Gildea, J. A. K. Howard and H. Puschmann, OLEX2: a complete structure solution, refinement and analysis program, *Journal of Applied Crystallography*, 2009, **42**, 339-341.
2. G. Sheldrick, Crystal structure refinement with SHELXL, *Acta Crystallographica Section C*, 2015, **71**, 3-8.
